# Blocking the autocrine regulatory loop of Gankyrin/STAT3/CCL24/CCR3 impairs the progression and pazopanib resistance of clear cell renal cell carcinoma

**DOI:** 10.1038/s41419-020-2306-6

**Published:** 2020-02-12

**Authors:** Chao Wang, Yuning Wang, Tianyu Hong, Bing Cheng, Sishun Gan, Linbao Chen, Jing Zhang, Li Zuo, Jian Li, Xingang Cui

**Affiliations:** 1 Department of Urinary Surgery, Gongli Hospital, Second Military Medical University (Naval Medical University), 219 Miaopu Road, Shanghai, 200135 China; 2 Department of Urology, the Affiliated Changzhou No. 2 People’s Hospital of Nanjing Medical University, 29 Xinglong Road, Changzhou, Jiangsu 213000 China; 3 Ningxia Medical University, Yinchuan, Ningxia 750004 China; 4 Department of Urology, Affiliated Hospital of Nantong University, 20 Xisi Road, Changzhou, Jiangsu China; 5 Department of Urinary Surgery, The Third Affiliated Hospital of Naval Medical University (Eastern Hepatobiliary Surgery Hospital), 700 North Moyu Road, Shanghai, 201805 China

**Keywords:** Oncogenes, Renal cell carcinoma

## Abstract

The poor prognosis of clear-cell renal cell carcinoma (ccRCC) patients is due to progression and targeted drug resistance, but the underlying molecular mechanisms need further elucidation. This study examined the biological function and related mechanisms of gankyrin in ccRCC based on the results of our previous study. To this end, in vitro functional experiments; in vivo models of subcutaneous tumor formation, lung metastasis, and orthotopic ccRCC; and antibody chip detection, co-IP, ChIP assays were performed to examine the biological role and molecular mechanisms of gankyrin in ccRCC. Two hundred fifty-six ccRCC patients were randomly divided into training and validation cohorts to examine the prognostic value of gankyrin and other markers through IHC and statistical analyses. We observed that the gankyrin-overexpressing ccRCC cell lines 786-O and 769-P exhibited increased proliferation, invasion, migration, tumorigenicity, and pazopanib resistance and decreased apoptosis, while gankyrin knockdown achieved the opposite results. Mechanistically, gankyrin recruited STAT3 via direct binding, and STAT3 binding to the CCL24 promoter promoted its expression. Reciprocally, an increase in autocrine CCL24 enhanced the expression of gankyrin and STAT3 activation via CCR3 in ccRCC, forming a positive autocrine-regulatory loop. Furthermore, in vivo experimental results revealed that blocking the positive loop through gankyrin knockdown or treatment with the CCR3 inhibitor SB328437 reversed the resistance to pazopanib and inhibited lung metastasis in ccRCC. Moreover, a positive correlation between gankyrin and STAT3 or CCL24 expression in ccRCC specimens was observed, and improved accuracy for ccRCC patient prognosis was achieved by combining gankyrin and STAT3 or CCL24 expression with existing clinical prognostic indicators, including the TNM stage and SSIGN score. In summary, targeting the gankyrin/STAT3/CCL24/CCR3 autocrine-regulatory loop may serve as a remedy for patients with advanced ccRCC, and combining gankyrin and STAT3 or CCL24 expression with the current clinical indicators better predicts ccRCC patient prognosis.

## Introduction

Renal cell carcinoma (RCC) serves as one of the most common malignant tumors worldwide^1^. Clear-cell RCC (ccRCC), which is the most common type of RCC, accounts for the majority of deaths related to RCC^1^. Although curative nephrectomy is the preferred treatment for localized ccRCC, many patients eventually experience progression and develop metastases^2^. In addition, although targeted therapies have been developed for advanced and metastatic ccRCC, the treatment responses vary, and many patients still experience progression^2^. However, the molecular mechanisms underlying this progression and treatment-induced drug resistance have not been well elucidated.

Gankyrin, also known as *PSMD10*, serves as an oncogene in various malignant cancers, including hepatocellular carcinoma, colorectal cancer, gastric cancer, prostate cancer, ovarian cancer, and cholangiocarcinoma^3–5^. Moreover, high gankyrin expression in specimens from patients with tumors indicates disease progression and poor prognosis^6^. In addition, gankyrin facilitates the proliferation, tumorigenicity, metastasis, and drug resistance of tumors through metabolic reprogramming and the mediation of signaling pathways such as the Wnt/β-catenin pathway, NF-κB pathway, PI3K/AKT/HIF-1α/cyclin D1, RhoA/ROCK, and mTORC1 signaling^6–9^. Our previous study demonstrated that gankyrin is upregulated in RCC specimens, and that high gankyrin expression in RCC patients predicts disease progression and poor prognosis^10^. However, the biological function and mechanisms of gankyrin in ccRCC have not been examined.

In addition to intratumoral oncogenes and related pathways, autocrine inflammatory factors and signaling can exert protumoral effects in tumors by interacting with the intratumoral pathway to form a positive regulatory loop^11,12^. Therefore, the identification of crucial autocrine inflammatory factors could aid the identification of potential targets and inhibitors to block the regulatory loop that continuously activates intratumoral pathways^13^. C–C motif chemokine ligand 24 (CCL24, also named eotaxin-2), a type of inflammatory chemokine, has been reported to be associated with various diseases, such as primary biliary cholangitis, allergies, and eosinophilic esophagitis^14,15^. Furthermore, CCL24 is strongly associated with primary and metastatic tumors of colorectal origin and the poor prognosis of patients, and serves as a potential target in immune therapy^16^. In addition, CCL24 is involved in acute promyelocytic leukemia (APL), and differentiation therapy with all-trans retinoic acid induces CCL24 production in the lung and APL cells, both of which trigger the migration of leukemic cells^17^. A recent study also demonstrated that CCL24 contributes to hepatocellular carcinoma malignancy through RhoB-VEGFA-VEGFR2 and indicates poor prognosis^18^. However, the expression and biological function of CCL24 in ccRCC have not been elucidated.

In this study, we mainly determined whether gankyrin facilitates the progression and targeted drug resistance of ccRCC, and examined whether the integration of gankyrin and STAT3 or CCL24 expression with established clinical indicators results in improved prediction of the prognosis of ccRCC patients.

## Materials and methods

### Cell culture

The ccRCC cell lines were bought from the Cell Bank of the Type Culture Collection of the Chinese Academy of Sciences (Shanghai, China) in 2018. The 786-O and 769-P cells were maintained in RPMI-1640 medium (Gibco, Waltham, MA, USA) supplemented with FBS (Gibco). The 786-O cells were continuously exposed to increasing doses of pazopanib (Selleck Chemicals, Houston, TX, USA) for ~21 weeks. The starting dose was 2 µM, and this dose was increased to 5 µM after 3 weeks, to 8 µM after 6 weeks, to 10 µM after 8 weeks, and maintained at 10 µM for 4 weeks. The established resistant 786-O cell lines were then maintained in the RPMI-1640 medium with 10% (v/v) FBS and 10 µM pazopanib. All the cells were cultured at 37 °C in 5% CO_2_, and the culture media was supplemented with 1% penicillin/streptomycin (Gibco). The cell lines were authenticated by short tandem repeat (STR) profiling, and the presence of mycoplasma contamination was detected using a Mycoplasma Detection Kit (Selleck Chemicals). The most recent tests were performed in June 2019. All ccRCC cell lines were cultured within 40 passages.

### Gene knockdown and overexpression

The short hairpin RNA (shRNA) interference vector pLKO.1-GFP, which contains a U6 promoter upstream of the shRNA, and the lentivirus packaging vectors pVSVG-I and pCMV-GAG-POL were obtained from Shanghai Integrated Biotech Solutions Co., Ltd. (Shanghai, China). The 786-O and 769-P cell lines were transduced with the shRNA-expressing lentivirus (sh-gankyrin) or control lentivirus. Seventy-two hours after transduction, the cells were observed and photographed under a microscope. Stable 786-O and 769-P cell lines in which gankyrin was knocked down were also generated using lentiviral constructs. The shRNA sequences are presented in Supplementary Table S1. The transfection of cells with gankyrin-overexpressing lentivirus vectors and STAT3-overexpressing lentivirus vectors were performed using the Lipofectamine 3000 reagent (L3000015, Invitrogen, Waltham, MA, USA) according to the manufacturer’s recommended protocol, and the sequences of the gankyrin-overexpressing lentivirus vectors and STAT3-overexpressing lentivirus vectors are shown in Supplementary Table S2. The small interfering RNAs (siRNAs) targeting STAT3, CCL24, CCR3, and the negative control siRNA (scrambled siRNA) were purchased from Shanghai Integrated Biotech Solutions Co., Ltd. (Shanghai, China). The transient transfection of siRNAs was performed with Lipofectamine 2000 (1168019, Invitrogen, Waltham, MA, USA) according to the manufacturer’s instructions. Twelve hours post-transfection, the supernatant was replaced with fresh medium containing 10% FBS. The siRNA sequences are presented in Supplementary Table S3.

### Cell proliferation, invasion, and migration assays

The proliferation of ccRCC cells under the indicated conditions was detected using a CCK-8 kit (CK-04, Dojindo, Kumamoto, Kyushu, Japan) according to the manufacturer’s instructions, as described in our previous study^19^. Prior to the assay, the medium was replaced with fresh medium, 10% v/v CCK-8 was added to each well, and the samples were incubated at 37 °C for 2 h. The OD values at an absorbance of 450 nm were then measured using a microplate reader (EXL800, BioTek Instruments, Winooski, VT, USA). The proliferation rates are presented as proportions of the control value, which was obtained from the control group. Invasion and migration assays were conducted in transwell chambers (Millipore, Billerica, MA, USA) with or without Matrigel (BD Biosciences, NJ, USA) according to the manufacturer’s instructions, as described in our previous study^20^. A total of 1 × 10^4^ cells were seeded in RPMI-1640 medium without fetal bovine serum (FBS) into the upper chamber of each uncoated transwell, and RPMI-1640 medium with 20% FBS and conditioned medium (CM) were placed in the lower chamber. Thirty-six hours after seeding, the noninvasive cells in the upper chamber were removed with a cotton swab, and the cells on the lower surface of the membrane were fixed with 4% paraformaldehyde fix solution (E672002, Sangon Biotech, Shanghai, China), stained with crystal violet (E607309, Sangon Biotech), and photographed at 200 × magnification. The data are presented as the means ± SDs from three independent experiments.

### Assessment of apoptosis

Apoptotic cells were evaluated through ANNXIN-V and PI staining (Invitrogen, A13201, USA) according to the manufacturer’s instructions, and then analyzed by flow cytometry with a Cyan ADP Sorter (Beckman, CA, USA).

### Western blot and coimmunoprecipitation assays

The western blot analysis was performed as described in our previous study^20^. The primary antibodies used in the this study were listed as follows: rabbit anti-gankyrin (ab182576), rabbit anti-STAT3 (ab32500), and rabbit anti-STAT3 (Phospho 727) (ab30647), rabbit anti-eotaxin-2 (CCL24) (ab203586), rabbit anti-CCR3 (ab32512) from Abcam (Cambridge, MA, USA) and rabbit anti-GAPDH (#2118S), rabbit anti-p44/42 MAPK (Erk1/2) (#4695), rabbit anti-p44/42 MAPK (Erk1/2) (Phospho Thr202/Tyr204) (#4370), rabbit anti-Akt (#9272), and rabbit anti-Akt (Phospho Ser473) (#9271) from Cell Signaling Technology (Danvers, MA, USA). The secondary antibody used in the assay was anti-rabbit IgG-HRP-linked antibody (#7074S) from Cell Signaling Technology. Coimmunoprecipitation analysis was performed according to previously published protocols^20^ using the above-mentioned antibodies.

### Real-time polymerase chain reaction

RT-PCR was performed as reported previously^20^. Briefly, RNAiso Plus (9109, Takara, Japan) and PrimeScript One Step RT reagent Kit (RR037B, Takara, Japan) were used for the extraction and reverse transcription of RNA. The results were normalized by GAPDH expression, and the fold change relative to the control group was calculated using the 2^−△△Ct^ method. The primer sequences are presented in Supplementary Table S4.

### Patients and samples

The paired tumor tissues and paracancerous tissue from 256 patients who were pathologically diagnosed with ccRCC between 2010 and 2015 were used to assess the expression of gankyrin, CCL24, and STAT3 in ccRCC in this study. The clinical data from these ccRCC patients at the time of diagnosis included the age, gender, World Health Organization/International Society of Urological Pathology grading system (WHO/ISUP grading)^21^, TNM stage, and SSIGN score, which are summarized in Supplementary Table S5. The ccRCC patients were randomly divided into the training set (*n* = 128) and the validation set (*n* = 128) at a 1:1 ratio. This study was performed following the recommendations for prognostic studies investigating tumor biomarkers (REMARK)^22^. In addition to the above-described cohorts, this study also included healthy individuals (*n* = 15), localized ccRCC patients (*n* = 50), and metastatic ccRCC patients (*n* = 20). All the experiments were approved by the institutional ethical review board of the hospital, and the written informed consents were obtained from all the patients.

### Immunohistochemistry (IHC)

The IHC assays were done as reported previously^20^. The primary antibodies used in the study were listed as follows: rabbit anti-gankyrin (ab182576), rabbit anti-STAT3 (ab32500), rabbit anti-eotaxin-2 (CCL24) (ab203586), rabbit anti-Ki-67 (ab15580), and rabbit anti-vimentin (ab137321) from Abcam. The presence of IHC staining for gankyrin, CCL24, and vimentin was scored semiquantitatively as negative (0), weakly positive (1 + ), moderately positive (2 + ), or strongly positive (3 + ), and the percentages of positive cells were also determined. For each observed tissue component, a summary value referred to as component H-Score was calculated by the multiplication of the intensity score, which ranged from 0 to 3, by the percentage of positive cells, which ranged from 0 to 300, and the total H-Score for a tissue section was derived as the sum of the component H-Scores weighted by the fraction of each component observed in the tissue section. IHC staining for STAT3 and Ki-67 were evaluated by the percentage of positive cells. To determine the number of stained cells in each observed tissue component, three respective areas of the tumor core were evaluated at ×400 magnification, and the mean value was adopted. The hematoxylin and eosin (H&E)-stained sections of the specimens were re-evaluated by two experienced pathologists using a double-blind procedure for the identification of representative areas.

### Antibody-microarray experiment and enzyme-linked immunosorbent assay (ELISA)

The antibody-microarray assay was performed as previously reported^20^. The cytokine profiles were measured with Quantibody Human Inflammatory Array 3 (RayBiotech, Norcross, GA, USA), which allowed the detection of 40 inflammation-associated cytokines. The CCL24 concentration in cell culture medium or blood serum was measured using an ELISA Kit for CCL24 (DCC240B, R&D Systems, Minneapolis, MN, USA) according to the manufacturer’s instructions.

### Nano-LC-ESI-MS/MS analysis

The Nano-LC-ESI-MS/MS analysis was performed as previously reported^20^. For the identified proteins reported here, the certainty should be > 98% if the identification is based on the LC-MS/MS sequencing of one peptide, and > 99.9% if it is based on the sequencing of two or more peptides.

### Chromatin immunoprecipitation (ChIP) and luciferase reporter assay

ChIP assays were performed according to our previously published study^20^. Primers complementary to the promoter region of CCL24 (forward primer: 5′-GGACTCTTATTGGCCGCCTTCC-3′; reverse primer: 5′-CGGGCATGGTGACTGGGATTTC-3′) were used for the detection of CCL24 genomic DNA, and primers specific to the human GAPDH promoter were used as the control (kit supplied). The enrichment of the targets was calculated as follows: fold enrichment = 2^(Ct [PDGFB-ChIP]−Ct [IgG])^. The STAT3-binding sites of the CCL24 promoter (sequence: CTGATGGAAA, -848 to -838 relative to the CCL24 transcription site) or its mutant sequence were cloned into a pGL3-basic luciferase reporter vector (Promega, USA). The ccRCC cells were cotransfected with 10 ng of the pTK-RL reporter control plasmid and 200 ng of pGL3-basic-CCL24-WT or pGL3-basic-CCL24-Mut using the Lipofectamine 3000 reagent (L3000015, Invitrogen, Waltham, MA, USA) according to the manufacturer’s recommended protocols. Cells were collected 48 h after transfection, and the CCL24 transcriptional activity was evaluated by measuring the luminescence using a Dual-Luciferase Assay Kit (E1910, Promega, Fitchburg, WI, USA). The fold-induction levels were derived relative to the normalized reporter activity.

### Animal experiments

For the subcutaneous tumor-formation assay, 786-O cells (5 × 10^6^ cells in 100 μl of PBS) subjected to different treatments were injected subcutaneously into the nude mice. The mice were euthanized 6 weeks after inoculation. All the subcutaneous tumors were removed and then fixed in 10% buffered formalin solution, which were used for further experiments.

The 786-O-PR cells were first transfected with the luciferase reporter gene, and then used in subcapsular renal tumor formation or lung metastasis assays. After the intraperitoneal injection of D-luciferin (150 mg/kg) (Gold Biotech, USA) in 100 μl of DPBS, the tumor growth was monitored weekly by live-animal bioluminescence optical imaging using an IVIS Lumina II imaging system (PerkinElmer, Hopkinton, MA, USA). Briefly, for renal subcapsular tumor cell implantation, 6-week-old male NOD-SCID mice were anesthetized and placed in the left lateral decubitus position. A vertical incision was made in the right flank through the skin and peritoneum to expose the lateral aspect of the kidney. The kidney was lifted gently and stabilized, and 50 μl of a Matrigel/medium (1:1) suspension containing 1 × 10^7^ 786-O-PR-luc cells from the indicated groups was inoculated under the renal capsule using a 24-gauge needle inserted from the lower pole of the kidney. Three weeks after tumor implantation, the mice were divided into four groups: the mice injected with 786-O-PR cells were treated with normal saline or pazopanib (80 mg/kg) in the absence or presence of CCR3 inhibitor (SB328437, TOCRIS, USA) (4 mg/kg), and the mice injected with gankyrin-knockdown 786-O-PR cells were treated with pazopanib (80 mg/kg). The mice were killed 8 weeks after injection, and the normal and injected kidneys were removed and fixed in 10% buffered formalin solution.

For the caudal vein injection of 6-week-old male NOD-SCID mice, 200 μl of PBS containing 1 × 10^7^ 786-O-PR-luc cells from the indicated groups was injected into the caudal vein of the mice using a 24-gauge needle. Three weeks after injection, the mice were divided into four groups: the mice injected with 786-O-PR cells were treated with normal saline or pazopanib (80 mg/kg) in the absence or presence of SB328437 (4 mg/kg), and the mice injected with gankyrin-knockdown 786-O-PR cells were treated with pazopanib (80 mg/kg). The mice were killed 8 weeks after injection, and the lung tumors were removed and then fixed in 10% buffered formalin solution for further experiments.

All experimental animal procedures were approved by the Animal Care and Use Committee of Gongli Hospital (Shanghai, China).

### Statistical analysis

The numerical data are expressed as the means ± SDs. The continuous variables were assayed using two-tailed Student’s *t* test or Wilcoxon test, and the categorical variables were investigated using a chi-square test or Fisher’s exact test. Time-dependent receiver-operating characteristic (ROC) analysis was performed using the “survivalROC” package to determine the optimal cutoff values for the H-scores of CCL24 and gankyrin. Survival curves were plotted using a Kaplan–Meier analysis and compared via the log-rank test. Variables with *p*-values < 0.05 in the univariate Cox proportional hazards analysis were included in the multivariate analysis. Differences were considered significant if *p* < 0.05. The performance of the prognostic prediction models was thoroughly measured. The prognostic accuracies of the CCL24 classifier and other prognostic indicators were indicated by Harrell’s concordance index using the “rms” package (C-index). A higher C-index indicates a better predictability of survival. All statistical analyses were performed using R software (version 3.5.2).

## Results

### Ectopic expression of gankyrin facilitates the proliferation, progression, pazopanib resistance, and tumorigenicity of clear-cell renal cell carcinoma (ccRCC)

**Fig. 1:**
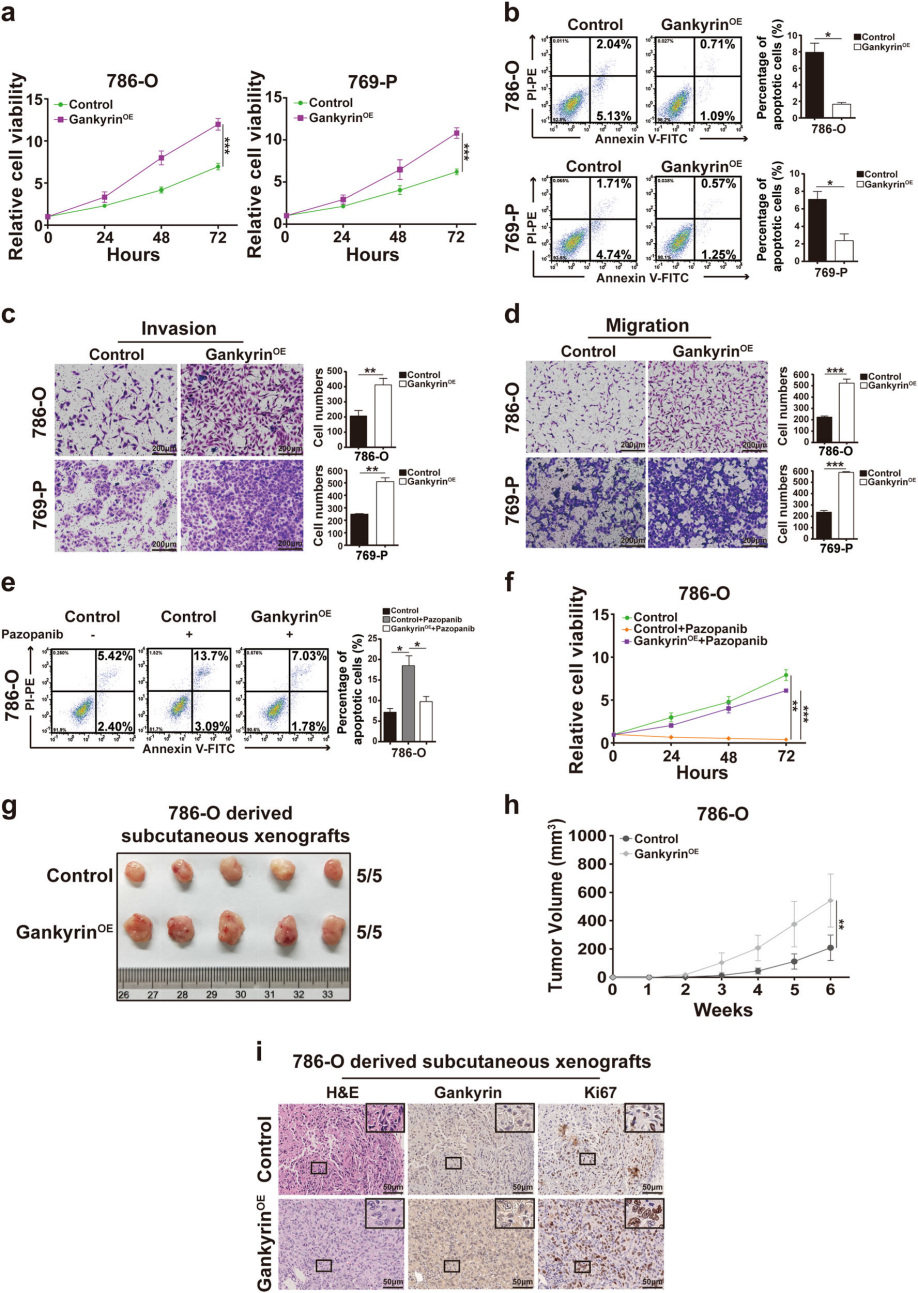
The ectopic expression of gankyrin facilitates the proliferation, progression, pazopanib resistance, and tumorigenicity of ccRCC. **a** CCK-8 assays were used to detect the proliferation of 786-O or 769-P cells with or without gankyrin overexpression at the indicated times, and the data are presented as fold changes relative to the control group. **b** Annexin V/propidium iodide (PI) double-staining of 786-O or 769-P cells with or without gankyrin overexpression was performed, and flow cytometry assays were employed to detect the percentage of apoptotic cells. **c**, **d** Representative images and the statistical analysis of the results from the invasion (**c**) or migration (**d**) assays with ccRCC cells are presented (scale bar = 200 µm). **e**, **f** Gankyrin-overexpressing and control 786-O cells were treated with pazopanib (5 μM) for 36 h, and the degree of apoptosis was examined by annexin V/PI staining. Cell viability was examined through CCK-8 experiments, and the data are presented as fold changes relative to the naïve control cells. **g**, **h** A total of 5 × 10^6^ gankyrin-overexpressing and control 786-O cells were subcutaneously injected into nude mice (*n* = 5/group), and the tumor xenografts derived from the two groups are presented (**g**). The volumes of the tumor xenografts from the two groups were compared at the indicated times (**h**). **i** Representative images of H&E and IHC staining for gankyrin and Ki-67 of subcutaneous xenografts from the two groups are shown (scale bar = 50 μm). All the data are presented as the means ± SDs, *P *<* 0.05, **P *<* 0.01, and ***P *<* 0.001.

Because our previous study demonstrated that gankyrin is commonly upregulated in RCC and predicts RCC patients’ poor prognosis^10^, we examined the biological role of gankyrin in ccRCC cells. The ccRCC cell lines (786-O and 769-P) were stably transfected with gankyrin (Supplementary Fig. S1a), and the proliferation activity of ccRCC cells was then determined through Cell Counting Kit-8 (CCK-8) proliferation experiments, which showed that the gankyrin-overexpressing ccRCC cells exhibited increased cell proliferation compared with the control cells (Fig. 1a). In addition, flow cytometry assays were employed to show that gankyrin overexpression induced fewer apoptotic events in populations of ccRCC cells compared with those observed in the control cells (Fig. 1b). Moreover, the effects of gankyrin overexpression on the invasion or migration abilities of ccRCC cells were examined through invasion or migration assays, respectively, and the results showed that the numbers of invaded and migrated ccRCC cells were higher in the gankyrin-overexpressing ccRCC cells compared with the control cells (Fig. 1c, d).

Because our previous study showed that the expression of gankyrin positively associates with the targeted drug resistance in RCC^10^, we also investigated the involvement of gankyrin in the resistance of ccRCC to the first-line targeted drug pazopanib. As presented in Fig. 1e, f, pazopanib induced fewer apoptotic events and increased proliferation in the population of gankyrin-overexpressing 786-O cells compared with those found in the control ccRCC cells, as detected by flow cytometry and CCK-8 assays, respectively. Furthermore, the subcutaneous xenograft model was used to evaluate the in vivo role of gankyrin in ccRCC growth. As shown in Fig. 1g, h, the gankyrin-overexpressing 786-O cell-derived xenografts exhibited a larger volume and faster growth than those derived from the control cells. In addition, immunohistochemistry (IHC) staining indicated that the gankyrin-overexpressing 786-O cell-derived xenografts exhibited higher Ki-67 expression than the control 786-O-derived tumor specimens (Fig. 1i; Supplementary Fig. S1b). These results suggest that gankyrin serves as an oncogene in ccRCC.

### Knockdown of gankyrin inhibits the proliferation, progression, pazopanib resistance, and tumorigenicity of ccRCC

**Fig. 2:**
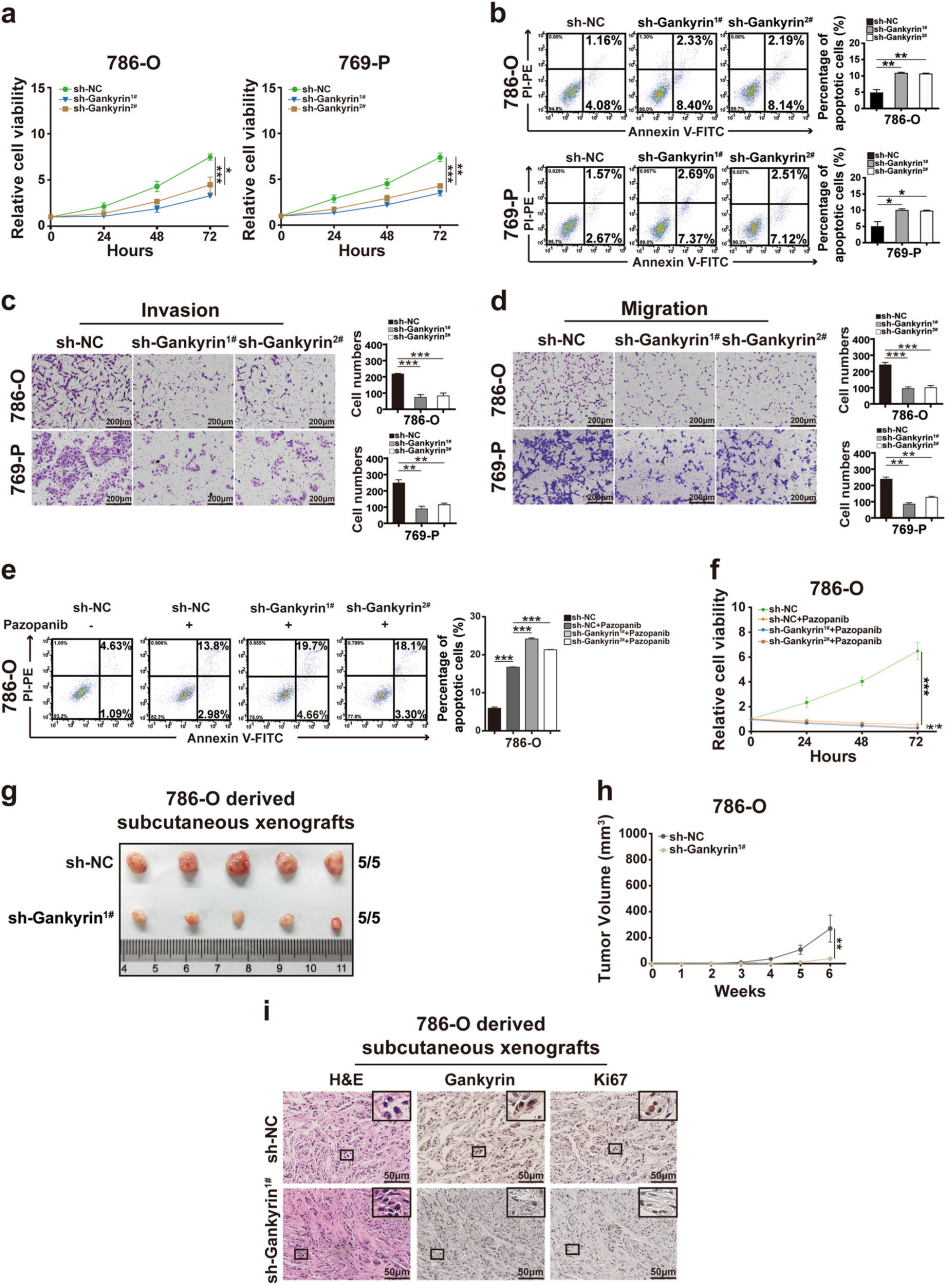
Knockdown of gankyrin inhibits the proliferation, progression, pazopanib resistance, and tumorigenicity of ccRCC. **a** CCK-8 assays were employed to examine the viability of 786-O or 769-P cells without or with gankyrin knockdown at the indicated times. The data are presented as fold changes relative to the control group. **b** The percentage of apoptosis among gankyrin-knockdown and control 786-O or 769-P cells was analyzed by Annexin V/PI double-staining and flow cytometry assays. **c**, **d** Representative images and the statistical analysis of the results from the invasion (**c**) or migration (**d**) assays with the ccRCC cells from different groups are presented (scale bar = 200 µm). **e**, **f** 786-O cells without or with gankyrin knockdown were treated with pazopanib (5 μM) for 36 h, and the resulting apoptosis was analyzed by flow cytometry assays. Cell viability was detected through CCK-8 assays, and the data are presented as fold changes relative to the naive control cells. **g**, **h** A total of 5 × 10^6^ 786-O cells without or with gankyrin knockdown were subcutaneously injected into nude mice (*n* = 5/group) and the tumor xenografts derived from the two groups are shown (**g**). The volumes of the tumor xenografts from the two groups were compared at the different indicated times (**h**). **i** Representative images of H&E and IHC staining for gankyrin and Ki-67 of subcutaneous xenografts from the two groups were shown (scale bar = 50 μm). All the data are presented as the means ± SDs, *P *<* 0.05, **P *<* 0.01, and ***P *<* 0.001.

To determine whether gankyrin is required for the growth and progression of ccRCC, short hairpin RNAs (shRNAs) were used to silence gankyrin in 786-O and 769-P cells (Supplementary Fig. S2a). CCK-8 and flow cytometry assays revealed that the gankyrin-knockdown ccRCC cells exhibited significantly decreased proliferation but increased apoptosis compared with the corresponding control cells (Fig. 2a, b). In addition, invasion or migration assays revealed less invaded and migrated cells in the populations of gankyrin-silenced ccRCC cells compared with their control cell populations (Fig. 2c, d). Contrary to the above-mentioned results for gankyrin overexpression, pazopanib treatment resulted in more apoptotic events and decreased proliferation in the gankyrin-knockdown 786-O cells compared with those found in the control cells (Fig. 2e, f), which suggests that gankyrin-knockdown ccRCC cells are sensitive to pazopanib. Furthermore, *i*n vivo assays demonstrated that gankyrin knockdown decreased the growth and volume of 786-O cell-derived subcutaneous xenografts in the mouse model (Fig. 2g, h). In addition, immunohistochemistry (IHC) staining indicated that the gankyrin-knockdown 786-O cell-derived xenografts exhibited lower Ki-67 expression than the control 786-O-derived tumor specimens (Fig. 2i; Supplementary Fig. S2b). Taken together, the results indicate that gankyrin plays a crucial role in the growth and progression of ccRCC.

### Gankyrin facilitates the growth and progression of ccRCC cells via CCL24/CCR3

**Fig. 3:**
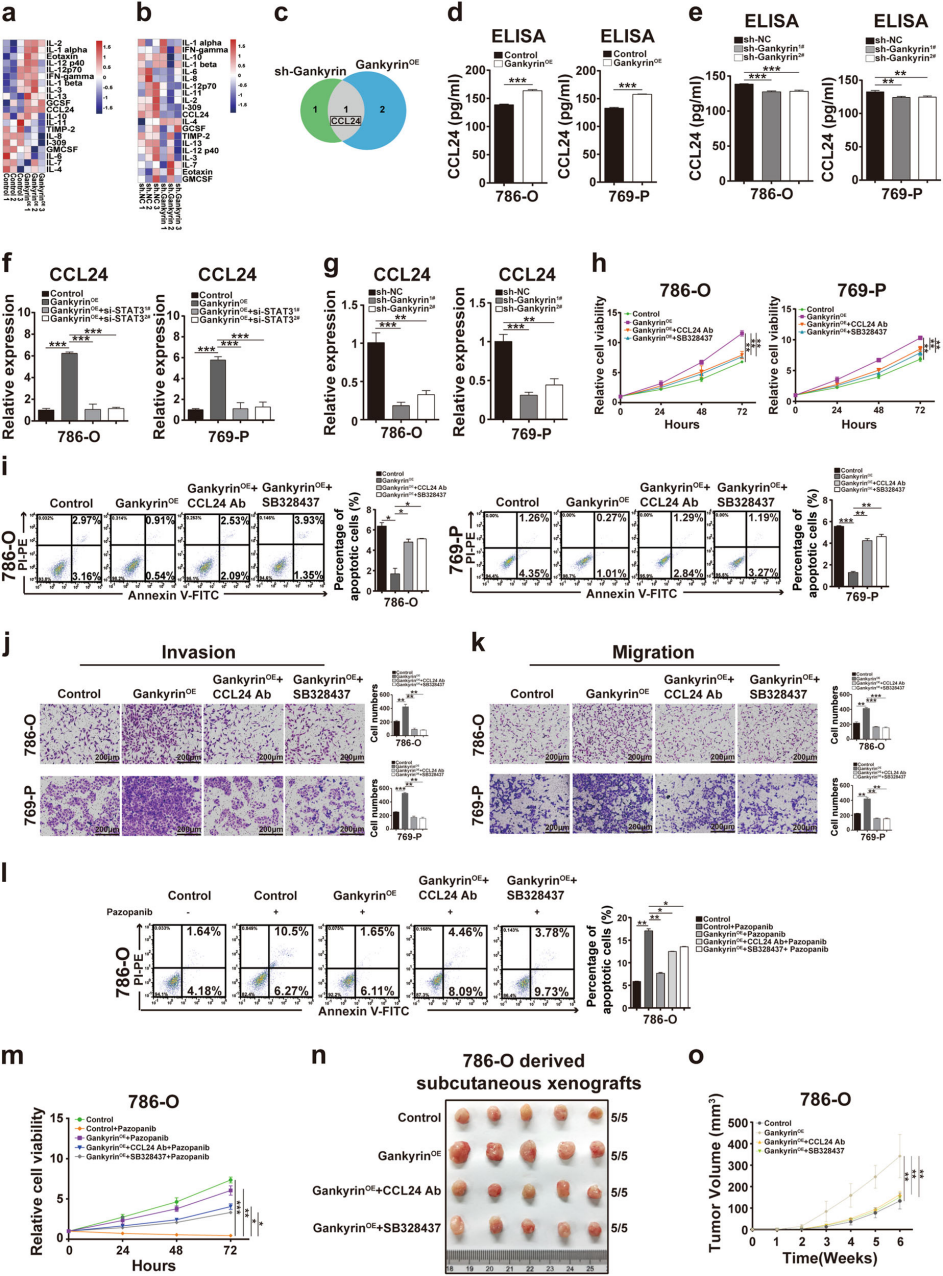
Gankyrin facilitates the growth and progression of ccRCC cells by promoting autocrine CCL24/CCR3. **a**, **b** Cytokine profiles of conditioned medium (CM) from 786-O cells with/without gankyrin overexpression (**a**) or with/without gankyrin knockdown (**b**) and control 786-O cells were analyzed using a RayBiotech Human Cytokine Antibody Array. Heatmaps of the significantly differentially expressed cytokines are presented. **c** A Venn diagram of the significantly differentially expressed cytokines among the indicated groups is shown. **d**, **e** ELISAs were performed to determine the CCL24 concentration in the CM from the gankyrin-overexpressing and control 786-O or 769-P cells (**d**) and from the 786-O or 769-P cells with or without gankyrin knockdown (**e**). **f** Real-time PCR was performed to determine the expression of *CCL24* mRNA in 786-O or 769-P cells with or without gankyrin overexpression in the absence and presence of STAT3 knockdown. **g** The mRNA expression of CCL24 in 786-O or 769-P cells with or without gankyrin knockdown was analyzed by real-time PCR. **h** CCK-8 assays were performed to determine the viability of 786-O or 769-P cells with or without gankyrin overexpression in the absence or presence of the CCL24 antibody (10 ng/ml) or SB328437 (10 ng/ml) at the indicated times. The data are presented as fold changes relative to the control group. **i** The percentage of apoptotic 786-O or 769-P cells with or without gankyrin overexpression in the absence or presence of the CCL24 antibody (10 ng/ml) or SB328437 (10 ng/ml) was analyzed by flow cytometry assays. **j**, **k** Representative images and statistical analysis of the results from the invasion (**j**) and migration (**k**) assays of 786-O and 769-P cells with or without gankyrin overexpression in the absence or presence of the CCL24 antibody (10 ng/ml) or SB328437 (10 ng/ml) are presented (scale bar = 200 µm). **l**, **m** 786-O cells with or without gankyrin overexpression in the absence or presence of the CCL24 antibody (10 ng/ml) or SB328437 (10 ng/ml) were treated with pazopanib (5 μM) for 36 h, and the resulting apoptosis was analyzed by flow cytometry assays. Cell viability was examined through CCK-8 assays. **n**, **o** A total of 5 × 10^6^ 786-O with or without gankyrin overexpression in the absence or presence of the CCL24 antibody (10 ng/ml) or SB328437 (10 ng/ml) were subcutaneously injected into nude mice (*n* = 5/group), and the tumor xenografts derived from the two groups are presented (**n**). The volumes of the tumor xenografts from the two groups were compared at the indicated times (**o**). All the data are presented as the means ± SDs, *P *<* 0.05, **P *<* 0.01, and ***P *<* 0.001.

Because autocrine signaling is often involved in the progression of ccRCC^23,24^, we subsequently investigated whether gankyrin regulates ccRCC by prompting tumor cells to secrete inflammatory factors. As shown in Fig. 3a, b, Supplementary Fig. S3a, b, and Supplementary Tables S6, 7, a RayBiotech Human Cytokine Antibody Array was employed to detect the cytokines that showed differential expression between the conditioned medium (CM) from gankyrin-overexpressing and gankyrin-knockdown 786-O cells and the CM from their respective control 786-O cells. The intersecting cytokines were then identified using a Venn plot, which demonstrated that CCL24 was unanimously expressed in these cells (Fig. 3c). Validation experiments based on enzyme-linked immunosorbent assays (ELISAs) confirmed that the amount of CCL24 secreted into the CM from gankyrin-overexpressing ccRCC cells was significantly higher than that secreted in the CM from control cells (Fig. 3d), whereas a lower CCL24 level was detected in the CM from gankyrin-knockdown ccRCC cells (Fig. 3e).

These findings prompted us to investigate whether CCL24 is responsible for the gankyrin-mediated growth and progression of ccRCC. First, real-time PCR results showed that the overexpression of gankyrin upregulated the mRNA expression of CCL24, while the knockdown of gankyrin achieved the opposite results (Fig. 3f, g). Second, in vitro assay results demonstrated that downregulation of CCL24 by siRNA or a neutralizing antibody alleviated the gankyrin-mediated promotion of the proliferation, invasion, migration, and pazopanib resistance ccRCC cells and the gankyrin-mediated inhibition of ccRCC cell apoptosis (Fig. 3h–m; Supplementary Fig. S3c–g). Furthermore, in vivo assays showed that the neutralizing antibody against CCL24 abated the gankyrin-mediated increases in the growth and volume of 786-O cell-derived subcutaneous xenografts (Fig. 3n, o) and resulted in lower gankyrin and Ki-67 expression, as demonstrated by IHC staining (Supplementary Fig. S3h). Thus, gankyrin-induced growth, invasion, migration, and drug resistance in ccRCC via CCL24.

Because CCL24 has been reported to usually activate downstream signaling pathways via C–C motif chemokine receptor 3 (CCR3)^18^ and because the most credible functional partner of CCL24 was also predicted as CCR3 by the STRING database (Supplementary Fig. S3i), we next examined whether blocking CCR3 could alleviate the gankyrin/CCL24-increased growth and progression of ccRCC. First, real-time PCR assay results demonstrated that gankyrin overexpression upregulated CCR3 expression in ccRCC cells (Supplementary Fig. S3j). However, when CCL24 was knocked down in ccRCC cells, the increased expression of CCR3 was abated (Supplementary Fig. S3j). Second, given that the binding of CCL24 to its receptor CCR3 often leads to the activation of downstream signaling such as ERK and AKT^25,26^. Western blot assays were performed to show that gankyrin overexpression increased the expressions of p-ERK and p-AKT in ccRCC cells (Supplementary Fig. S3k). However, the addition of a neutralizing antibody for CCL24 or an inhibitor of CCR3, SB328437^27^ alleviated the role of gankyrin overexpression in up-regulating p-ERK and p-AKT (Supplementary Fig. S3k). Furthermore, knockdown of CCR3 by siRNA or SB328437 weakened the enhanced proliferation, invasion, migration, and tumorigenicity in ccRCC cells mediated by gankyrin and inhibited the gankyrin-mediated apoptosis of ccRCC cells (Fig. 3h–o; Supplementary Fig. S3c–g and S3l). These results indicate that gankyrin facilitates the growth and progression of ccRCC cells via CCL24/CCR3.

### CCL24 plays a protumoral role in ccRCC, and high CCL24 expression in specimens from ccRCC patients predicts poor postoperative prognosis

**Fig. 4:**
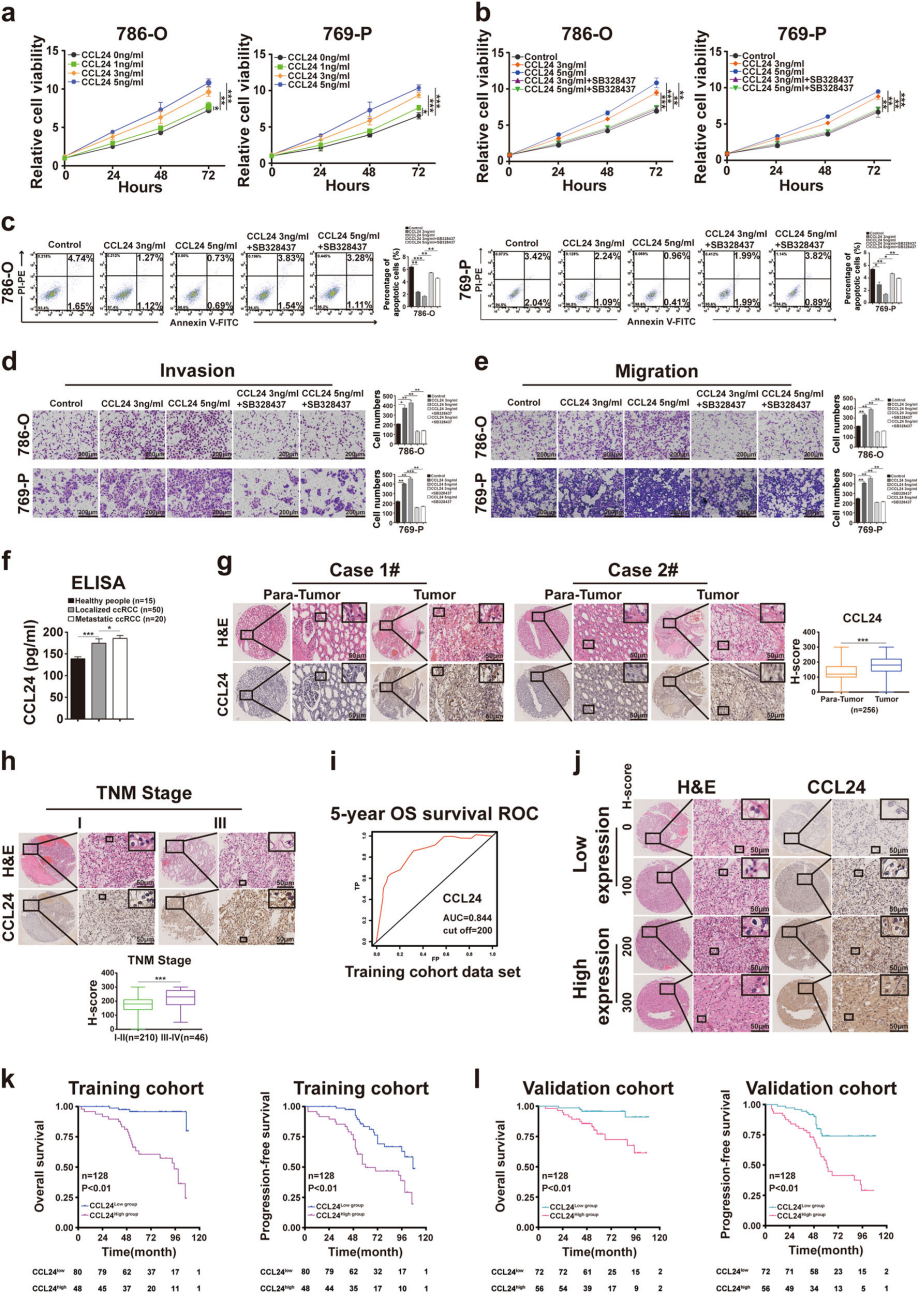
CCL24 exerts a protumoral role in ccRCC, and high CCL24 expression in ccRCC patients predicts poor postoperative prognosis. **a** 786-O or 769-P cells were exposed to a range of concentrations of human recombinant CCL24 protein (1, 3, and 5 ng/ml) for 3 days, and the viability of the ccRCC cells was determined by CCK-8 assays. **b** 786-O or 769-P cells were treated with human recombinant CCL24 protein (3 or 5 ng/ml) in the absence or presence of SB328437 (10 ng/ml) for 3 days, and the viability of the cells was determined using CCK-8 assays. The data are presented as fold changes relative to the naive group. **c** The percentage of apoptotic 786-O or 769-P cells treated with human recombinant CCL24 protein (3 or 5 ng/ml) in the absence or presence of SB328437 (10 ng/ml) for 3 days was analyzed by flow cytometry assays. **d**, **e** Representative images and the statistical analysis of the results from the invasion (**d**) and migration (**e**) assays of 786-O or 769-P cells treated with human recombinant CCL24 protein (3 or 5 ng/mL) in the absence or presence of SB328437 (10 ng/ml) for 3 days (scale bar = 200 µm) are shown. **f** The CCL24 concentration in the blood serum of healthy individuals (*n* = 15) and patients with localized ccRCC (*n* = 50), and metastatic ccRCC (*n* = 20) was determined by ELISA. **g** Representative images of H&E and IHC staining for CCL24 of ccRCC tissues and matched adjacent tissues are presented (*n* = 256; scale bar = 50 μm). **h** Representative images of H&E and IHC for CCL24 staining and statistical charts in ccRCC patients with high (*n* = 46) and low (*n* = 210) TNM stages are presented (scale bar = 50 μm). **i** A time-dependent receiver-operating characteristic (ROC) analysis was performed to examine the optimal H-score cutoff value for CCL24 in the training cohort (*n* = 128). **j** Representative images of H&E and IHC staining for CCL24 in ccRCC specimens are shown (scale bar = 50 µm). **k**, **l** Kaplan–Meier analyses of the OS and PFS of ccRCC patients were performed with the training cohort (*n* = 128) (**k**), and the validation cohort (*n* = 128) (**l**) (*p-*value: log-rank test). All the data are presented as the means ± SDs, *P *<* 0.05, **P *<* 0.01, and ***P *<* 0.001.

Given the crucial role of CCL24 in gankyrin-facilitating ccRCC, we subsequently investigated its biological function and clinical significance in ccRCC. First, ccRCC cells were treated with human recombinant CCL24 protein, and CCK-8 proliferation assays were performed to show that recombinant CCL24 enhanced the proliferation of ccRCC cells in a dose-dependent manner (Fig. 4a, b). Moreover, ccRCC cells with recombinant CCL24 exhibited less apoptosis and increased invasion and migration abilities relative to the naive ccRCC cells (Fig. 4c–e). However, the addition of the CCR3 inhibitor SB328437 to the CM of recombinant CCL24-treated ccRCC cells abated both the enhancement in the malignant proliferation, invasion, and migration abilities of ccRCC cells and the decreased apoptosis of these cells (Fig. 4b–e). Therefore, CCL24 exerts protumoral effects on ccRCC cells via CCR3.

We subsequently examined whether the CCL24 expression level in specimens from ccRCC patients is associated with progression and prognosis. First, CCL24 was upregulated in the blood serum of patients with localized ccRCC compared with healthy individuals, and the highest concentration of CCL24 was observed in patients with metastatic ccRCC (Fig. 4f), which indicated that the CCL24 level in blood serum is positively associated with disease progression. Moreover, IHC was performed for the detection of CCL24 expression in postoperative ccRCC specimens (*n* = 256). Most ccRCC tissue specimens displayed increased CCL24 expression compared with matched adjacent tissues (214/256; *p* *<* 0.001) (Fig. 4g). In addition, the expression of CCL24 in ccRCC patients with TNM stage III–IV was higher than that in patients with a lower stage (*p* *<* 0.001) (Fig. 4h). To further examine whether CCL24 expression is predictive of ccRCC patients’ prognosis, the ccRCC patients (*n* = 256) were first randomly divided into the training and validation cohorts at a 1:1 ratio. Then a time-dependent receiver-operating characteristic (ROC) analysis was performed to determine the optimal cutoff value of CCL24 expression (H-score) for dividing ccRCC patients in the training cohort. As shown in Fig. 4i, the optimal cutoff value was 200, with an area under the curve (AUC) of 0.844 using 5-year overall survival (OS) as the endpoint. Thus, using the cutoff value, we divided ccRCC patients in the training cohort into the CCL24^high^ and CCL24 ^low^ groups (Fig. 4i, j; Supplementary Table S8). As presented in Supplementary Table S8 and Fig. 4k, the CCL24^high^ group exhibited a higher TNM stage (*p* < 0.05) and a SSIGN (*p* < 0.01) score compared with the CCL24^low^ group, and a Kaplan–Meier survival analysis demonstrated that the CCL24^high^ subgroup experienced worse OS (*p* < 0.001) and progression-free survival (PFS) (*p* < 0.001). Moreover, these results were confirmed in the validation and the combined cohorts (Fig. 4l; Fig. S4a; Supplementary Tables S9 and 10). Taken together, these results suggest that high CCL24 expression predicts poor prognosis of ccRCC patients.

### Gankyrin/STAT3/CCL24/CCR3 forms a positive autocrine-regulatory loop in ccRCC

**Fig. 5:**
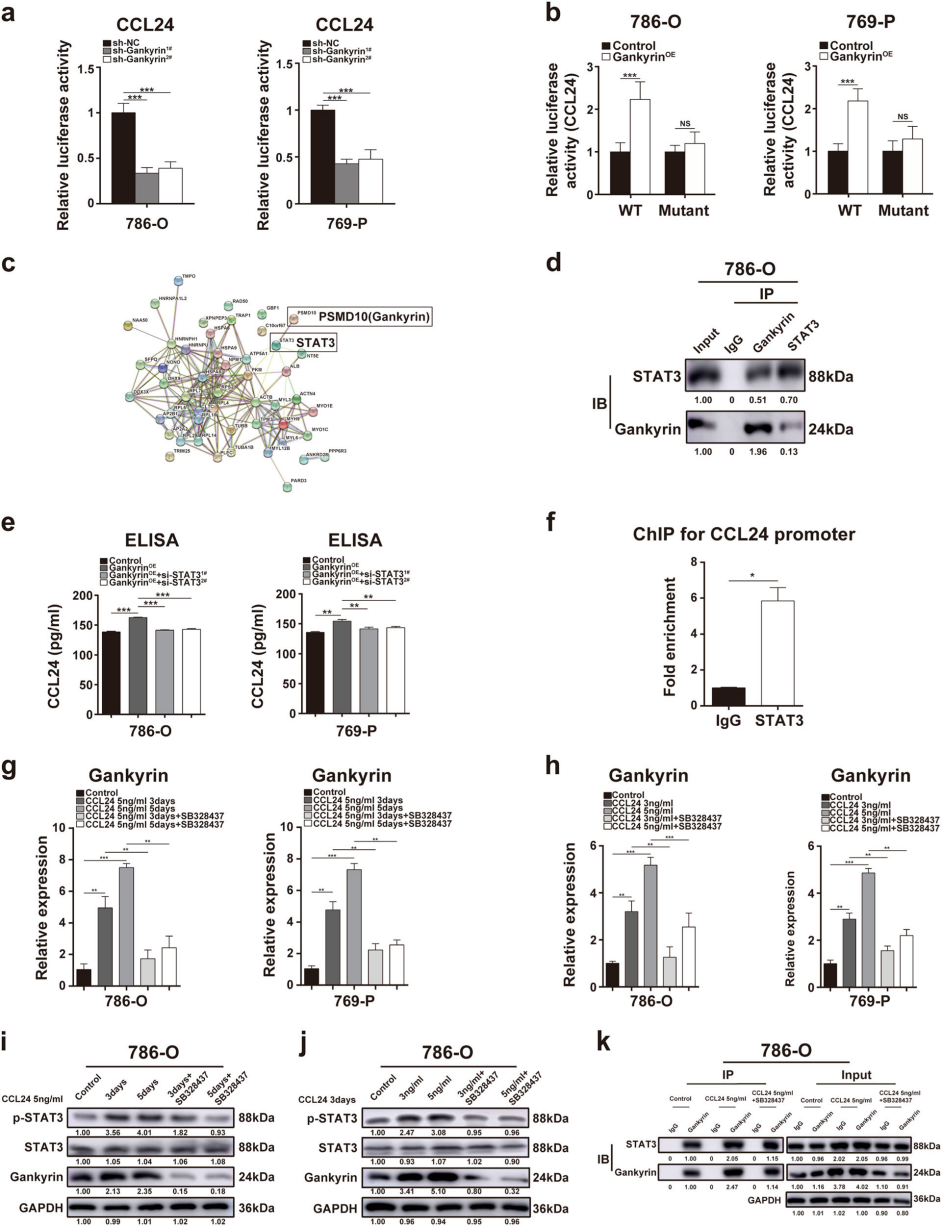
Gankyrin/STAT3/CCL24/CCR3 forms a positive autocrine-regulatory loop in ccRCC. **a** Luciferase assays were used to determine the transcriptional activity of CCL24 in 786-O or 769-P cells with or without gankyrin knockdown. **b** The STAT3-binding sites in the CCL24 promoter in 786-O or 769-P cells were blocked using reporter constructs harboring mutant STAT3 variants, and luciferase assays were performed to determine the transcriptional activity of CCL24 in 786-O or 769-P cells with or without gankyrin overexpression in the presence of the wild-type or mutant STAT3 plasmid. **c** Gankyrin-interacting proteins were identified by nano-LC-ESI-MS/MS, and the STRING protein–protein interaction network is presented. **d** Western blot assays revealed that endogenous gankyrin coimmunoprecipitated (co-IP) with endogenous STAT3 in 786-O cells. IgG served as the control for co-IP. **e** An ELISA was performed to determine the concentration of CCL24 in the CM from 786-O or 769-P cells with or without gankyrin overexpression in the absence and presence of STAT3 knockdown. **f** A ChIP-PCR analysis was performed to determine the binding of STAT3 to the promoter of CCL24 in 786-O cells. **g** Real-time PCR was used to determine the expression of *PSMD10* (gankyrin) mRNA in 786-O or 769-P cells treated with human recombinant CCL24 protein (5 ng/ml) for 3 and 5 days in the absence or presence of SB328437 (10 ng/ml). **h** Real-time PCR assays were performed to examine the expression of *PSMD10* (gankyrin) mRNA in 786-O or 769-P cells treated with human recombinant CCL24 protein (3, 5 ng/ml) for 3 days in the absence or presence of SB328437 (10 ng/ml). **i** Western blot assays were used to detect the protein expression of gankyrin, p-STAT3, and STAT3 in 786-O cells treated with human recombinant CCL24 protein (5 ng/ml) for 3 and 5 days in the absence or presence of SB328437 (10 ng/ml). **j** Western blot assays were performed to detect the protein expression of gankyrin, p-STAT3, and STAT3 in 786-O cells treated with human recombinant CCL24 protein (3, 5 ng/ml) for 3 days in the absence or presence of SB328437 (10 ng/ml). **k** Immunoprecipitation assays were employed to examine the binding of gankyrin to STAT3 in 786-O cells treated with human recombinant CCL24 protein (5 ng/ml) for 3 days in the absence or presence of SB328437 (10 ng/ml). All the data are presented as the means ± SDs, *P *<* 0.05, **P *<* 0.01, and ***P *<* 0.001.

We subsequently investigated the molecular mechanisms through which gankyrin regulates CCL24 in ccRCC. First, gankyrin-knockdown ccRCC cells exhibited decreased CCL24 transcriptional activity, but ccRCC cells overexpressing gankyrin presented increased CCL24 transcription, as determined by luciferase assays (Fig. 5a, b). Because gankyrin is not a common transcription factor, nanoscale liquid chromatography tandem electrospray ionization mass spectrometry (nano-LC-ESI-MS/MS) was performed to determine whether gankyrin recruited any other transcription factor to facilitate CCL24 transcription (Supplementary Table S11). Among the gankyrin-interacting proteins, STAT3, which serves as a transcription factor in tumor progression, was significantly expressed^28,29^ (Fig. 5c; Supplementary Table S11). Coimmunoprecipitation (Co-IP) assays confirmed a direct interaction between endogenous gankyrin and STAT3 in 786-O cells (Fig. 5d).

Then real-time PCR and ELISA assays were performed, the results of which indicated that STAT3 knockdown alleviated the gankyrin-mediated increase in CCL24 mRNA expression in ccRCC cells and CCL24 concentration in conditional medium of ccRCC cells (Figs. 3f and 5e; Supplementary Fig. S5a). Moreover, STAT3 was overexpressed in gankyrin-knockdown ccRCC cells, which rescued the decreased mRNA expression of CCL24 induced by gankyrin-knockdown in ccRCC cells (Supplementary Fig. S5b). In addition, STAT3 overexpression increased the expression of CCL24 in ccRCC cells, whereas STAT3 knockdown decreased CCL24 expression. Thus, gankyrin upregulates CCL24 expression via STAT3 (Supplementary Fig. S5c-e). Furthermore, JASPAR software (http://jaspar.genereg.net) was used for the prediction of putative transcription factor binding sites on the CCL24 promoter (Supplementary Fig. S5f), and chromatin immunoprecipitation (ChIP) assays using antibodies against STAT3 demonstrated that STAT3 bound to the CCL24 promoter in 786-O cells (Fig. 5f). The binding sites of the CCL24 promoter bound by STAT3 were blocked using reporter constructs harboring mutated variants, and luciferase assays showed no increase in CCL24 transcriptional activity in the ccRCC cells relative to that in the control cells (Fig. 5b). Therefore, gankyrin facilitates the expression and transcription of CCL24 via STAT3 in ccRCC.

We then examined whether autocrine CCL24/CCR3 reciprocally regulates gankyrin/STAT3 in ccRCC. First, upregulated expression of both gankyrin mRNA and protein was observed in the ccRCC cells treated with recombinant CCL24 compared with the naive ccRCC cells (Fig. 5g–j; Supplementary Fig. S5g, h). In addition, the phosphorylation of STAT3 was increased in CCL24-treated ccRCC cells (Fig. 5i, j; Supplementary Fig. S5g, h). However, the small-molecule CCR3 antagonist SB328437 abated the increases in the expression of gankyrin and p-STAT3 induced by recombinant CCL24 (Fig. 5g–j; Supplementary Fig. S5g, h). Furthermore, IP assays showed that recombinant CCL24 treatment enhanced the binding of gankyrin to STAT3 in ccRCC, whereas SB328437 inhibited this interaction (Fig. 5k; Supplementary Fig. S5i). These results indicate that gankyrin/STAT3/CCL24/CCR3 forms a positive autocrine-regulatory loop in ccRCC cells.

### Blocking the positive autocrine-regulatory loop ameliorates pazopanib resistance and inhibits lung metastasis of ccRCC

**Fig. 6:**
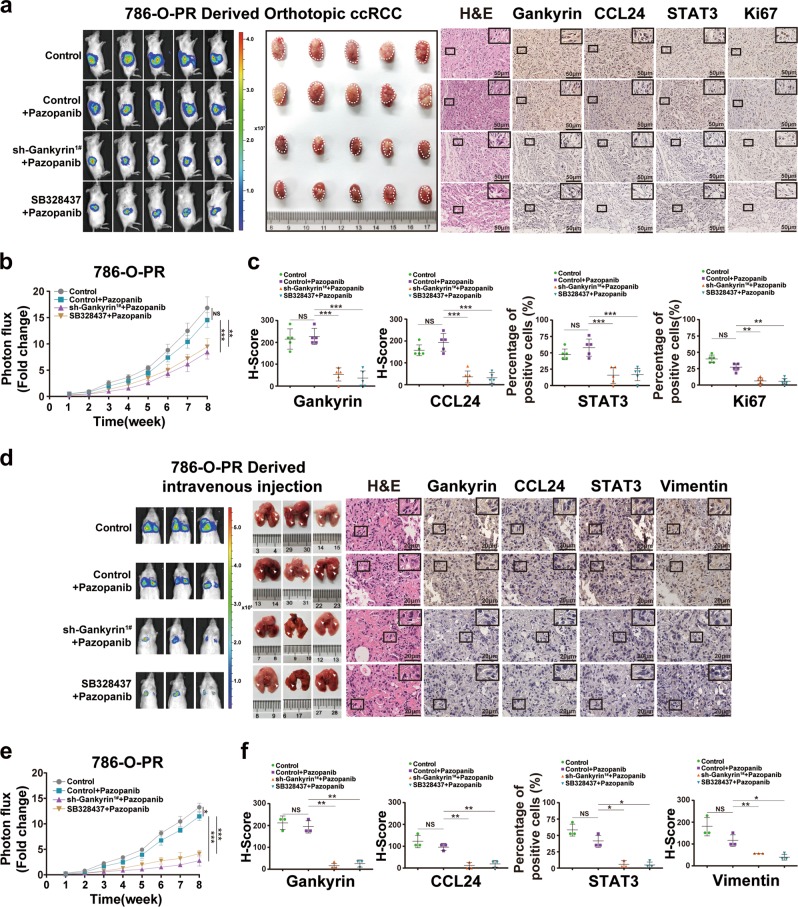
Blocking the positive autocrine-regulatory loop ameliorates pazopanib resistance and inhibits lung metastasis of ccRCC. **a** Images of the luciferase intensity and orthotopic xenografts from different groups are presented (*n* = 5/group). The photon flux levels in the different groups of mice were examined, and the results are presented as the fold increases in tumor growth. **b** Representative images of H&E and IHC staining for gankyrin, STAT3, CCL24, and Ki-67 in tumor specimens from mice in the four groups were shown (scale bar = 50 µm). **c** The results from the IHC staining for gankyrin, STAT3, and CCL24 were evaluated through the H-score, and the results from the IHC staining for Ki-67 were evaluated by the percentage of positive cells (scale bar = 50 µm). **d** Images of the luciferase intensity and lung metastases from the different groups are presented (*n* = 3/group). The photon flux levels in the different groups of mice were examined, and the results are presented as fold increases in tumor growth and lung metastases. **e** Representative images of H&E and IHC staining for gankyrin, STAT3, CCL24, and vimentin in lung metastases from mice in the four groups were presented (scale bar = 20 µm). **f** The IHC staining scores for gankyrin, STAT3, CCL24, and vimentin in lung metastases from mice in the different groups are presented (scale bar = 20 µm). All the data are presented as the means ± SDs, *P *<* 0.05, **P *<* 0.01, and ***P *<* 0.001.

Based on the above findings, we next investigated whether blocking the regulatory loop by knocking down gankyrin or inhibiting CCR3 serves as an effective strategy for inhibiting the pazopanib resistance of ccRCC in vivo. An orthotopic ccRCC model was established through the injection of luciferase-expressing pazopanib-resistant 786-O (786-O-PR) cells (the pazopanib resistance was confirmed as shown in Supplementary Fig. S6a, c) without or with gankyrin knockdown (gankyrin-KD) into the subcapsular kidney of NOD/SCID mice. Three weeks postinjection, all the mice were randomly divided into four subgroups: mice injected with naive 786-O-PR cells, mice injected with pazopanib-treated 786-O-PR cells, mice injected with gankyrin-knockdown 786-O-PR cells that were treated with pazopanib, and mice injected with 786-O-PR cells that were treated with the CCR3 inhibitor SB328437. As shown in Fig. 6a, b, no significant difference in tumor growth was observed between the pazopanib-treated 786-O-PR and naive 786-O-PR groups, which indicated the resistant features of 786-O-PR-derived orthotopic ccRCC in vivo. However, the gankyrin-knockdown and SB328437-treated 786-O-PR groups with pazopanib treatment exhibited reduced tumor growth compared with that obtained with the pazopanib-treated and naive 786-O-PR groups (Fig. 6a, b). In addition, IHC assays showed that the orthotopic xenografts derived from the 786-O-PR group with gankyrin knockdown or SB328437 treatment exhibited lower STAT3, CCL24, and Ki-67 expression compared with the other two groups (Fig. 6b, c). Therefore, blocking the positive regulatory loop consisting of gankyrin/STAT3/CCL24/CCR3 through gankyrin knockdown or treatment with the CCR3 inhibitor reverses the pazopanib resistance of ccRCC in vivo.

Moreover, luciferase-expressing 786-O-PR cells without or with gankyrin knockdown were injected into the caudal vein of NOD/SCID mice, and 3 weeks postinjection, all the mice were randomly divided into four subgroups: mice injected with naive 786-O-PR cells, mice injected with 786-O-PR cells that had been treated with pazopanib, mice injected with gankyrin-knockdown 786-O-PR cells that had been treated with pazopanib, and mice injected with 786-O-PR cells that had been treated with SB328437. No significant difference in lung metastases was found between the pazopanib-treated 786-O-PR and naive 786-O-PR groups (Fig. 6d, e). However, the gankyrin-knockdown 786-O-PR and SB328437-treated 786-O-PR groups with pazopanib treatment exhibited reduced lung metastases compared with the pazopanib-treated and naive 786-O-PR groups (Fig. 6d, e**)**. In addition, IHC assays showed that the lung metastases obtained with gankyrin knockdown or SB328437 treatment exhibited lower vimentin, STAT3, and CCL24 expression compared with the other two groups (Fig. 6e, f). Thus, blocking the positive regulatory loop suppresses the lung metastasis of ccRCC.

### The combination of gankyrin, STAT3, or CCL24, and established indicators yields superior prognostic accuracy in predicting the prognosis of ccRCC patients

**Fig. 7:**
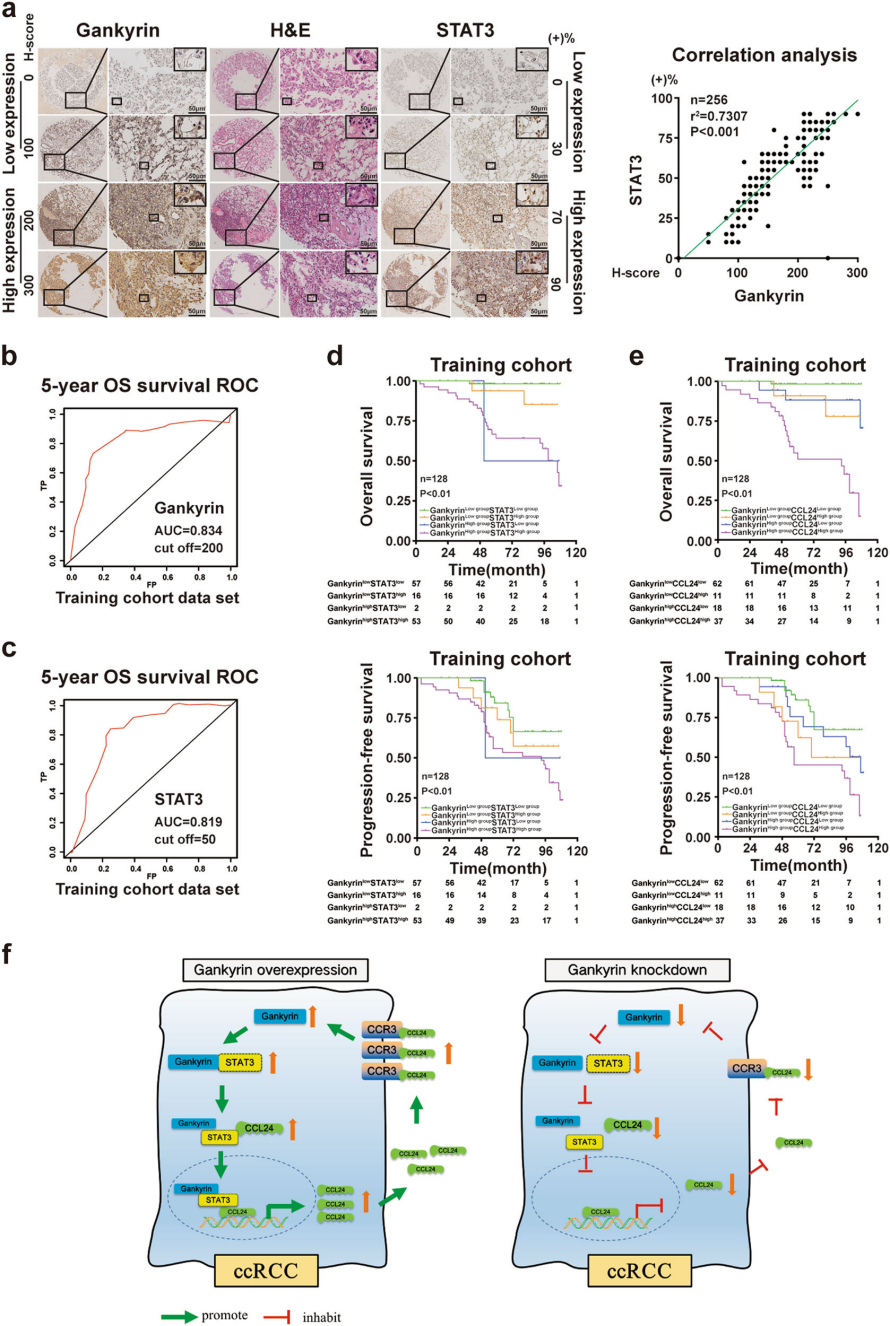
The combination of gankyrin, STAT3 or CCL24, and established indicators yields superior prognostic accuracy in predicting the prognosis of ccRCC patients. **a** Representative images of H&E staining and IHC staining for gankyrin and STAT3 in ccRCC tissues are presented (scale bar = 50 µm), and the results from the correlation analysis between gankyrin and STAT3 expression in the ccRCC samples are shown. **b**, **c** A time-dependent ROC curve analysis with the training cohort was performed to examine the optimal H-score cutoff value for gankyrin or STAT3 (*n* = 128). **d** According to the H-scores for gankyrin and STAT3 in ccRCC specimens, the patients were divided into four groups. Kaplan–Meier analyses of the OS and PFS of ccRCC patients in the training cohort are shown. **e** According to the H-scores for gankyrin and CCL24 in ccRCC specimens, the patients were divided into four groups. Kaplan–Meier analyses of the OS and PFS of ccRCC patients in the training cohort are presented. **f** Schematic diagram of the underlying mechanisms described in our study and the clinical significance of our findings. All the data are presented as the means ± SDs, *P *<* 0.05, **P *<* 0.01, and ***P *<* 0.001.

Given the close regulation between gankyrin and STAT3 or CCL24 in the progression of ccRCC, we further examined whether the combination of gankyrin and STAT3 or CCL24 expression in ccRCC patients is predictive of disease progression and prognosis. First, a correlation analysis demonstrated that gankyrin expression was positively correlated with STAT3 expression or CCL24 expression in ccRCC specimens (Fig. 7a; Supplementary Fig. S7a). Second, according to the optimal cutoff values for gankyrin and STAT3 or CCL24 (Figs. 7b, c and 4i), the ccRCC patients were divided into four groups. As shown in Supplementary Tables S12–17, high expression levels of both gankyrin and STAT3 or CCL24 predicted a high TNM stage and a high SSIGN score (all *p* < 0.05). Furthermore, a Kaplan–Meier survival analysis showed that the gankyrin^high^STAT3^high^ group or gankyrin^high^CCL24^high^ group experienced the worst OS and PFS rates, whereas the gankyrin^low^STAT3^low^ or gankyrin^low^CCL24^low^ group experienced the best OS and PFS rates (all *p* < 0.01) (Fig. 7d, e). The above findings were confirmed with validation and combined cohorts (all *p* < 0.01) (Supplementary Fig. S7b, e).

To further evaluate the prognostic value of gankyrin and STAT3 or CCL24 in ccRCC patients, univariate and multivariate Cox regression analyses were performed. As shown in Supplementary Tables S18–23, gankyrin expression, STAT3 or CCL24 expression, TNM stage, and SSIGN were revealed as independent risk factors with the training set, and the significance of these factors was confirmed with the validation and combined cohorts (all *p* < 0.05). We subsequently compared the prognostic accuracy of gankyrin and STAT3 or CCL24 with that of established indicators, namely, the TNM stage and SSIGN score, in predicting the prognosis of ccRCC patients. As shown in Table 1 and Supplementary Table S24, through a time-dependent concordance index (C-index) analysis using the training set, we found that the combination of gankyrin and STAT3 or CCL24 yielded a higher C-index value for predicting the OS and PFS of ccRCC patients compared with the values obtained with gankyrin, STAT3 or CCL24 alone. Moreover, the incorporation of both gankyrin and STAT3 or CCL24 with the clinical indicators TNM stage and SSIGN score yielded the highest C-index value among all the groups (Table 1; Supplementary Table S24). These findings were also confirmed with the validation and combined cohorts (Table 1; Supplementary Table S24). Taken together, the results suggest that an improved prognostic accuracy for ccRCC patients can be accomplished by combining gankyrin and STAT3 or CCL24 with existing clinical prognostic indicators.

**Table 1:** C-index analysis of the prognostic accuracy of gankyrin, STAT3 and other variables for OS and PFS in the training, validation, and combined cohorts.

	Overall survival			Progression-free survival		
Index (95% CI)	Training cohort (*n* = 128)	Validation cohort (*n* = 128)	Combine cohort (*n* = 256)	Training cohort (*n* = 128)	Validation cohort (*n* = 128)	Combined cohort (*n* = 256)
TNM stage	0.7225 (0.6433–0.7617)	0.7138 (0.6344–0.7633)	0.7334 (0.6654–0.7815)	0.6823 (0.6113–0.7209)	0.6674 (0.6011–0.7069)	0.6787 (0.6141–0.7133)
SSIGN	0.7159 (0.6367–0.7551)	0.7013 (0.6218–0.7408)	0.7205 (0.6513–0.7697)	0.6837 (0.6149–0.7252)	0.6637 (0.6043–0.7188)	0.6704 (0.6089–0.7206)
Gankyrin	0.7519 (0.6330–0.8608)	0.7617 (0.6437–0.8834)	0.7942 (0.6880–0.9061)	0.7387 (0.6302–0.8359)	0.7138 (0.6179–0.8271)	0.7277 (0.6241–0.8305)
STAT3	0.7234 (0.6266–0.7689)	0.7211 (0.6170–0.7752)	0.7301 (0.6327–0.7875)	0.7089 (0.6201–0.7577)	0.6912 (0.6137–0.7687)	0.7051 (0.6185–0.7618)
Gankyrin + STAT3	0.8054 (0.6611–0.8716)	0.8055 (0.6747–0.8663)	0.8183 (0.7188–0.9278)	0.7622 (0.6780–0.8364)	0.7535 (0.6614–0.8263)	0.7589 (06684–0.8326)
TNM stage + SSIGN	0.7825 (0.6521–0.8929)	0.7779 (0.6461–0.8898)	0.8086 (0.6945–0.9226)	0.7461 (0.6454–0.8407)	0.7210 (0.6336–0.8313)	0.7342 (0.6431–0.8354)
Gankyrin + STAT3 + TNM stage	0.8520 (0.7328–0.9412)	0.8461 (0.7237–0.9484)	0.8569 (0.7477–0.9561)	0.8130 (0.6810–0.8949)	0.8068 (0.6842–0.8894)	0.8077 (0.6813–0.8941)
Gankyrin + STAT3 + SSIGN	0.8471 (0.7299–0.9343)	0.8393 (0.7174–0.9311)	0.8535 (0.7412–0.9457)	0.8134 (0.6826–0.8991)	0.7971 (0.6704–0.8837)	0.8041 (0.6803–0.8857)

## Discussion

It is well known that the elucidation of molecular mechanisms and the identification of applicable prognostic factors are critical for the treatment and follow-up of patients with ccRCC in clinical practice. However, there remain a lack of effective treatment targets and comprehensive reliable prognostic markers for ccRCC patients. Based on our previous studies, this study further demonstrated that gankyrin facilitated the progression of ccRCC by activating the positive regulatory loop consisting of STAT3/CCL24/CCR3. Furthermore, blocking the regulatory loop achieved effective inhibition of ccRCC, and the integration of gankyrin and CCL24 with established indicators yielded superior accuracy in predicting the postoperative prognosis of ccRCC patients.

The oncogene gankyrin has been reported in some malignant tumors, and increased gankyrin expression in tissues indicates disease progression and short survival in tumor patients^30^. A recent study of ours also indicated that gankyrin is commonly upregulated in RCC specimens and serves as an independent risk factor for PFS and OS in RCC patients^10^. Many studies have elucidated the intratumoral signaling pathways regulated by gankyrin, and among these, gankyrin activates STAT3 by mediating IL-6 signaling or binding to Src homology 2 domain-containing protein tyrosine phosphatase-1 (SHP-1)^5,31^. However, whether gankyrin directly regulates STAT3 signaling has not yet been examined. This study demonstrated that gankyrin directly binds to STAT3 in ccRCC cells, as determined by nano-LC-ESI-MS/MS and confirmed by co-IP assays. We found that the transcription factor STAT3, which is recruited by gankyrin, directly binds to the promoter of CCL24 and triggers its transcription in ccRCC. In addition, although some previous studies have shown that the long noncoding RNA Linc-GALH promotes gankyrin/AKT signaling by controlling the methylation status of gankyrin or KIFC1, the upstream regulators should be further studied to determine an effective inhibitor for gankyrin signaling in tumors^32^. Our study found that autocrine CCL24 can trigger the expression and transcription of gankyrin in ccRCC via CCR3, which has not been previously reported.

CCL24, a member of the CC class of chemokines, has been shown to play a protumoral role in several malignant tumors, such as hepatocellular carcinoma and colon cancer^18,33^. Although its receptor CCR3 is upregulated in human renal cancer specimens and is correlated with the grade of malignancy^34^, the expression, prognostic value, and biological function of CCL24 in ccRCC have not been examined. Because autocrine signaling is often involved in the progression of ccRCC^23,24^, we focused on tumor-secreted factors by performing cytokine array-based analysis comparing gankyrin-overexpressing and gankyrin-knockdown tumor cells with wild-type control cells. This analysis revealed that CCL24 was the most consistently altered factor among all the cytokines in all independent tumor clones. In addition, CCL24 expression was commonly increased in ccRCC specimens, and ccRCC patients with high CCL24 expression exhibited poor OS and PFS. Moreover, CCL24 expression was positively associated with gankyrin expression in ccRCC samples, and the combination of gankyrin and CCL24 expression with the TNM stage or SSIGN score achieved better accuracy in predicting ccRCC patient survival.

Many studies have recently demonstrated that the progression of malignant tumors depends not only on the tumor itself but also on the microenvironment, which can sustain and facilitate the survival and evolution of tumors^35,36^. Our previous studies have also shown that the combination of targeting tumors and tumor-associated macrophages (TAMs) achieved better therapeutic effects than the inhibition of tumors^21,37^. Hence, although this study preliminarily elucidated the molecular mechanisms through which gankyrin regulates the progression of ccRCC (Fig. 7f), our further study will determine whether gankyrin can recruit inflammatory immune cells and mediate antitumoral immunity in ccRCC, and these findings will provide new therapeutic strategies for advanced or metastatic ccRCC.

## Supplementary information

Supplementary Tables S1-S24

Supplementary Figure Legend

Supplementary Figure S1

Supplementary Figure S2

Supplementary Figure S3

Supplementary Figure S4

Supplementary Figure S5

Supplementary Figure S6

Supplementary Figure S7
