# Supplementary material for: Blocking the autocrine regulatory loop of Gankyrin/STAT3/CCL24/CCR3 impairs the progression and pazopanib resistance of clear cell renal cell carcinoma
Source: Cell Death Dis. 2020 Feb 12;11(2):117. doi: 10.1038/s41419-020-2306-6 (PMC7015941; doi:10.1038/s41419-020-2306-6)
Supplement: Supplementary file 1 — Supplementary Tables S1-S24 [file 41419_2020_2306_MOESM1_ESM.docx]

**Supplementary Tables S1-S24**

**Table S1 Sequences of shRNAs for gankyrin**

| **Primer-NC-F** | gatcTGTTCTCCGAACGTGTCACGTTTCAAGAGAACGTGACACGTTCGGAGAATTTTTTc |
| --- | --- |
| **Primer-NC-R** | aattgAAAAAATTCTCCGAACGTGTCACGTTCTCTTGAAACGTGACACGTTCGGAGAACa |
| **Primer-F1** | GATCCGGGCTAATCCAGATGCTAAGGTTCAAGAGACCTTAGCATCTGGATTAGCCCTTTTTTG |
| **Primer-R1** | AATTCAAAAAAGGGCTAATCCAGATGCTAAGGTCTCTTGAACCTTAGCATCTGGATTAGCCCG |
| **Primer-F2** | GATCCGGAGAGTATTCTGGCCGATAATTCAAGAGATTATCGGCCAGAATACTCTCCTTTTTTG |
| **Primer-R2** | AATTCAAAAAAGGAGAGTATTCTGGCCGATAATCTCTTGAATTATCGGCCAGAATACTCTCCG |
| **Primer-F3** | GATCCGATAAAGACGATGCAGGTTGGTTCAAGAGACCAACCTGCATCGTCTTTATCTTTTTTG |
| **Primer-R3** | AATTCAAAAAAGATAAAGACGATGCAGGTTGGTCTCTTGAACCAACCTGCATCGTCTTTATCG |

**Table S2 Sequences of gankyrin overexpressed lentivirus vectors and STAT3 overexpressed lentivirus vectors**

| **PGMLV-CMV-6×his-H_PSMD10-EF1-ZsGreen1-T2A-Puro** | GAATTCGCCACCATGCATCATCACCATCACCATGAGGGGTGTGTGTCTAACCTAATGGTCTGCAACCTGGCCTACAGCGGGAAGCTGGAAGAGTTGAAGGAGAGTATTCTGGCCGATAAATCCCTGGCTACTAGAACTGACCAGGACAGCAGAACTGCATTGCACTGGGCATGCTCAGCTGGACATACAGAAATTGTTGAATTTTTGTTGCAACTTGGAGTGCCAGTGAATGATAAAGACGATGCAGGTTGGTCTCCTCTTCATATTGCGGCTTCTGCTGGCCGGGATGAGATTGTAAAAGCCCTTCTGGGAAAAGGTGCTCAAGTGAATGCTGTCAATCAAAATGGCTGTACTCCCTTACATTATGCAGCTTCGAAAAACAGGCATGAGATCGCTGTCATGTTACTGGAAGGCGGGGCTAATCCAGATGCTAAGGACCATTATGAGGCTACAGCAATGCACCGGGCAGCAGCCAAGGGTAACTTGAAGATGATTCATATCCTTCTGTACTACAAAGCATCCACAAACATCCAAGACACTGAGGGTAACACTCCTCTACACTTAGCCTGTGATGAGGAGAGAGTGGAAGAAGCAAAACTGCTGGTGTCCCAAGGAGCAAGTATTTACATTGAGAATAAAGAAGAAAAGACACCCCTGCAAGTGGCCAAAGGTGGCCTGGGTTTAATACTCAAGAGAATGGTGGAAGGTTAAGGATCC |
| --- | --- |
| **pcDNA3.0-STAT3** | ATGGCCCAATGGAATCAGCTACAGCAGCTTGACACACGGTACCTGGAGCAGCTCCATCAGCTCTACAGTGACAGCTTCCCAATGGAGCTGCGGCAGTTTCTGGCCCCTTGGATTGAGAGTCAAGATTGGGCATATGCGGCCAGCAAAGAATCACATGCCACTTTGGTGTTTCATAATCTCCTGGGAGAGATTGACCAGCAGTATAGCCGCTTCCTGCAAGAGTCGAATGTTCTCTATCAGCACAATCTACGAAGAATCAAGCAGTTTCTTCAGAGCAGGTATCTTGAGAAGCCAATGGAGATTGCCCGGATTGTGGCCCGGTGCCTGTGGGAAGAATCACGCCTTCTACAGACTGCAGCCACTGCGGCCCAGCAAGGGGGCCAGGCCAACCACCCCACAGCAGCCGTGGTGACGGAGAAGCAGCAGATGCTGGAGCAGCACCTTCAGGATGTCCGGAAGAGAGTGCAGGATCTAGAACAGAAAATGAAAGTGGTAGAGAATCTCCAGGATGACTTTGATTTCAACTATAAAACCCTCAAGAGTCAAGGAGACATGCAAGATCTGAATGGAAACAACCAGTCAGTGACCAGGCAGAAGATGCAGCAGCTGGAACAGATGCTCACTGCGCTGGACCAGATGCGGAGAAGCATCGTGAGTGAGCTGGCGGGGCTTTTGTCAGCGATGGAGTACGTGCAGAAAACTCTCACGGACGAGGAGCTGGCTGACTGGAAGAGGCGGCAACAGATTGCCTGCATTGGAGGCCCGCCCAACATCTGCCTAGATCGGCTAGAAAACTGGATAACGTCATTAGCAGAATCTCAACTTCAGACCCGTCAACAAATTAAGAAACTGGAGGAGTTGCAGCAAAAAGTTTCCTACAAAGGGGACCCCATTGTACAGCACCGGCCGATGCTGGAGGAGAGAATCGTGGAGCTGTTTAGAAACTTAATGAAAAGTGCCTTTGTGGTGGAGCGGCAGCCCTGCATGCCCATGCATCCTGACCGGCCCCTCGTCATCAAGACCGGCGTCCAGTTCACTACTAAAGTCAGGTTGCTGGTCAAATTCCCTGAGTTGAATTATCAGCTTAAAATTAAAGTGTGCATTGACAAAGACTCTGGGGACGTTGCAGCTCTCAGAGGATCCCGGAAATTTAACATTCTGGGCACAAACACAAAAGTGATGAACATGGAAGAATCCAACAACGGCAGCCTCTCTGCAGAATTCAAACACTTGACCCTGAGGGAGCAGAGATGTGGGAATGGGGGCCGAGCCAATTGTGATGCTTCCCTGATTGTGACTGAGGAGCTGCACCTGATCACCTTTGAGACCGAGGTGTATCACCAAGGCCTCAAGATTGACCTAGAGACCCACTCCTTGCCAGTTGTGGTGATCTCCAACATCTGTCAGATGCCAAATGCCTGGGCGTCCATCCTGTGGTACAACATGCTGACCAACAATCCCAAGAATGTAAACTTTTTTACCAAGCCCCCAATTGGAACCTGGGATCAAGTGGCCGAGGTCCTGAGCTGGCAGTTCTCCTCCACCACCAAGCGAGGACTGAGCATCGAGCAGCTGACTACACTGGCAGAGAAACTCTTGGGACCTGGTGTGAATTATTCAGGGTGTCAGATCACATGGGCTAAATTTTGCAAAGAAAACATGGCTGGCAAGGGCTTCTCCTTCTGGGTCTGGCTGGACAATATCATTGACCTTGTGAAAAAGTACATCCTGGCCCTTTGGAACGAAGGGTACATCATGGGCTTTATCAGTAAGGAGCGGGAGCGGGCCATCTTGAGCACTAAGCCTCCAGGCACCTTCCTGCTAAGATTCAGTGAAAGCAGCAAAGAAGGAGGCGTCACTTTCACTTGGGTGGAGAAGGACATCAGCGGTAAGACCCAGATCCAGTCCGTGGAACCATACACAAAGCAGCAGCTGAACAACATGTCATTTGCTGAAATCATCATGGGCTATAAGATCATGGATGCTACCAATATCCTGGTGTCTCCACTGGTCTATCTCTATCCTGACATTCCCAAGGAGGAGGCATTCGGAAAGTATTGTCGGCCAGAGAGCCAGGAGCATCCTGAAGCTGACCCAGGCGCTGCCCCATACCTGAAGACCAAGTTTATCTGTGTGACACCAACGACCTGCAGCAATACCATTGACCTGCCGATGTCCCCCCGCACTTTAGATTCATTGATGCAGTTTGGAAATAATGGTGAAGGTGCTGAACCCTCAGCAGGAGGGCAGTTTGAGTCCCTCACCTTTGACATGGAGTTGACCTCGGAGTGCGCTACCTCCCCCATGTGA |

**Table S3 Sequences of siRNAs for STAT3, CCL24, CCR3**

| **Control** | **Primer-NC-F** | UUCUCCGAACGUGUCACGUTT | **Primer-NC-R** | ACGUGACACGUUCGGAGAATT |
| --- | --- | --- | --- | --- |
| **STAT3** | **Primer-F1** | CCAACGACCUGCAGCAAUAUU | **Primer-R1** | UAUUGCUGCAGGUCGUUGGUU |
|  | **Primer-F2** | GCCUCUCUGCAGAAUUCAAAC | **Primer-R2** | UUGAAUUCUGCAGAGAGGCUG |
|  | **Primer-F3** | GAGUUGAAUUAUCAGCUUAAA | **Primer-R3** | UAAGCUGAUAAUUCAACUCAG |
| **CCL24** | **Primer-F1** | CAGUUGCUGUUCUCAAAUAAU | **Primer-R1** | UAUUUGAGAACAGCAACUGUG |
|  | **Primer-F2** | GCAUGUUCUUUGUUUCCAAGA | **Primer-R2** | UUGGAAACAAAGAACAUGCAG |
|  | **Primer-F3** | GAGUGAUCUUCACCACCAAGA | **Primer-R3** | UUGGUGGUGAAGAUCACUCCU |
| **CCR3** | **Primer-F1** | GAAUUAUGACCAACAUCUA | **Primer-R1** | UAGAUGUUGGUCAUAAUUC |
|  | **Primer-F2** | GCAUGAACCCGGUGAUCUA | **Primer-R2** | UAGAUCACCGGGUUCAUGC |
|  | **Primer-F3** | GCUGAUACCAGAGCACUGA | **Primer-R3** | UCAGUGCUCUGGUAUCAGC |

**Table S4 The primer Sequences**

| **Gankyrin-F** | 5′-AACTGACCAGGACAGCAGAACT-3′ |
| --- | --- |
| **Gankyrin-R** | 5′-ACAGCATTCACTTGAGCACCTT-3′ |
| **CCL24-F** | 5′-GGAGTGGGTCCAGAGGTACAT-3′ |
| **CCL24-R** | 5′-CAGGTGGTTTGGTTGCCAG-3′ |
| **CCR3-F** | 5′-GTCATCATGGCGGTGTTTTTC-3′ |
| **CCR3-R** | 5′-CAGTGGGAGTAGGCGATCAC-3′ |
| **GAPDH-F** | 5′-GGCATCCTGGGCTACACTGA-3′ |
| **GAPDH-R** | 5′-GAGTGGGTGTCGCTGTTGAA-3′ |

**Table S5 Demographics and characteristics of patients with ccRCC.**

| **Characteristic** | **training cohort (n=128)** | **validation cohort (n=128)** | **combined cohort (n=256)** |
| --- | --- | --- | --- |
| **Age** |  |  |  |
| <60 | 80 | 68 | 148 |
| ≥60 | 48 | 60 | 108 |
| **Gender** |  |  |  |
| Male | 101 | 78 | 179 |
| Female | 27 | 50 | 77 |
| **WHO/ISUP grade** |  |  |  |
| I-II | 102 | 101 | 203 |
| III-IV | 26 | 27 | 53 |
| **TNM stage** |  |  |  |
| I-II | 104 | 106 | 210 |
| III-IV | 24 | 22 | 46 |
| **SSIGN** |  |  |  |
| 0-4 | 106 | 104 | 210 |
| ≥5 | 22 | 24 | 46 |

**Table S6 Expressions of cytokines in CM from 786-O cells with and without gankyrin overexpression detected**

|  | **OE.Gankyrin.1** | **OE.Gankyrin.2** | **OE.Gankyrin.3** | **Control.1** | **Control.2** | **Control.3** | **foldchange** | **pvalue** | **mean.case** | **mean.control** |
| --- | --- | --- | --- | --- | --- | --- | --- | --- | --- | --- |
| IL-2 | 398.7268462 | 404.3369013 | 372.462184 | 292.9320217 | 238.0364961 | 325.8955957 | 1.371893061 | 0.039889352 | 391.8419772 | 285.6213712 |
| IL-1 alpha | 313.6511148 | 322.5231211 | 263.6304355 | 225.8623795 | 193.9965873 | 246.1627439 | 1.351014024 | 0.032251206 | 299.9348905 | 222.0072369 |
| GCSF | 141.5303063 | 120.0433547 | 153.7351845 | 73.3478507 | 125.8057609 | 93.0927236 | 1.421091714 | 0.098647597 | 138.4362818 | 97.41544507 |
| GMCSF | 47562.58546 | 32957.62909 | 41661.17149 | 53978.04616 | 55783.72658 | 45056.45184 | 0.789192528 | 0.117516733 | 40727.12868 | 51606.07486 |
| IL-1 beta | 305.7737323 | 329.2475414 | 243.4238893 | 198.758894 | 231.8803798 | 272.171856 | 1.24990218 | 0.155656508 | 292.8150543 | 234.2703766 |
| IL-3 | 283.323192 | 355.3980648 | 251.5774079 | 188.6525095 | 196.3643244 | 275.5828871 | 1.347712747 | 0.138902438 | 296.7662216 | 220.199907 |
| IL-8 | 29605.69809 | 30898.83574 | 25010.62296 | 34284.83732 | 34933.43429 | 35118.41275 | 0.819607766 | 0.069659834 | 28505.05226 | 34778.89479 |
| TIMP-2 | 17796.71392 | 20476.73146 | 18196.76289 | 24890.03422 | 20943.42328 | 27075.62781 | 0.774529101 | 0.074463414 | 18823.40276 | 24303.02844 |
| IFN-gamma | 602.3571849 | 625.8691918 | 497.6009698 | 344.8420872 | 386.7303816 | 631.6092575 | 1.266028816 | 0.311298066 | 575.2757822 | 454.3939088 |
| Eotaxin | 316.8020678 | 293.3839666 | 275.683463 | 229.9968095 | 257.9254871 | 275.5828871 | 1.160266513 | 0.084850063 | 295.2898325 | 254.5017279 |
| IL-13 | 136.8038768 | 123.031986 | 121.4756108 | 121.1234862 | 111.5993387 | 106.3104691 | 1.124702147 | 0.097649438 | 127.1038245 | 113.011098 |
| I-309 | 258.1155679 | 261.6297597 | 229.5983577 | 289.2569728 | 266.4493405 | 285.3896015 | 0.890913476 | 0.076129795 | 249.7812284 | 280.3653049 |
| IL-6 | 36434.60101 | 28357.37845 | 31987.90789 | 44554.30205 | 35115.2765 | 32937.48475 | 0.859447751 | 0.293350182 | 32259.96245 | 37535.68777 |
| IL-12 p40 | 895.3958154 | 886.2536885 | 761.3495722 | 762.419517 | 727.6845139 | 829.8754398 | 1.096129991 | 0.238868575 | 847.6663587 | 773.3264902 |
| IL-11 | 1978.929784 | 1873.996313 | 1758.56035 | 1881.012522 | 1939.492326 | 2265.493151 | 0.922032253 | 0.326183478 | 1870.495482 | 2028.666 |
| IL-12p70 | 303.8043866 | 293.3839666 | 263.6304355 | 251.5877217 | 242.2984227 | 299.4601048 | 1.085048035 | 0.36020838 | 286.9395962 | 264.4487497 |
| IL-7 | 908.3934965 | 1071.175246 | 982.5580775 | 1055.504666 | 1064.37672 | 976.5497768 | 0.956626085 | 0.468865175 | 987.3756067 | 1032.143721 |
| CCL24 | 582.521 | 569.5725 | 592.415 | 329.2231294 | 370.6297698 | 341.2452357 | 1.67564271 | 0.000404199 | 581.5028333 | 347.0327116 |
| IL-4 | 298.684088 | 325.5117524 | 293.4085035 | 395.3740094 | 336.5343565 | 224.4174207 | 0.959510197 | 0.822707002 | 305.8681146 | 318.7752622 |
| IL-10 | 325.8610577 | 291.516072 | 246.2598958 | 312.2260283 | 236.6158539 | 307.1349247 | 1.008949096 | 0.943025956 | 287.8790085 | 285.3256023 |

**by RayBio Human Cytokine Antibody Array
Table S7 Expressions of cytokines in CM from 786-O cells with and without gankyrin knockdown detected**

|  | **sh.Gankyrin.1** | **sh.Gankyrin.2** | **sh.Gankyrin.3** | **sh.NC.1** | **sh.NC.2** | **sh.NC.3** | **foldchange** | **pvalue** | **mean.case** | **mean.control** |
| --- | --- | --- | --- | --- | --- | --- | --- | --- | --- | --- |
| IL-6 | 21942.23745 | 19942.33844 | 27616.3195 | 33659.66667 | 40341.21368 | 21160.75413 | 0.730345751 | 0.264866644 | 23166.96513 | 31720.54483 |
| IL-8 | 27211.6018 | 24863.42481 | 29466.19208 | 32613.16667 | 40815.35281 | 27297.79141 | 0.809532464 | 0.240470151 | 27180.40623 | 33575.43696 |
| IL-12p70 | 290.1197511 | 248.2350613 | 245.7390209 | 266.1666667 | 326.8498378 | 352.2203251 | 0.829521035 | 0.159681923 | 261.3646111 | 315.0789432 |
| IL-13 | 113.7303758 | 134.6885889 | 107.8988851 | 134.1666667 | 145.6343581 | 146.4268862 | 0.835979626 | 0.084996423 | 118.7726166 | 142.0759703 |
| I-309 | 192.697882 | 175.258 | 170.4029707 | 222.6666667 | 232.5184922 | 272.003857 | 0.740328637 | 0.036419981 | 179.4529509 | 242.3963386 |
| IL-2 | 269.5195321 | 244.686734 | 286.7185207 | 295.1666667 | 295.0750687 | 346.0130984 | 0.85545597 | 0.103940967 | 266.9749289 | 312.0849446 |
| TIMP-2 | 5248.764136 | 8417.371497 | 4688.910245 | 7875.666667 | 6884.864279 | 6990.133058 | 0.843884392 | 0.434902734 | 6118.348626 | 7250.221335 |
| IL-10 | 313.2949975 | 228.7192613 | 274.7144248 | 316.1666667 | 297.5574726 | 316.4094018 | 0.8780768 | 0.258879218 | 272.2428945 | 310.0445137 |
| Eotaxin | 223.1690393 | 293.0326929 | 226.6980412 | 216.6666667 | 189.8211463 | 254.8146139 | 1.123388851 | 0.410751718 | 247.6332578 | 220.4341423 |
| IL-4 | 358.538 | 339.389 | 325.4876 | 105.6666667 | 244.4340306 | 254.8146139 | 1.691831205 | 0.095820694 | 341.1382 | 201.6384371 |
| IL-11 | 2062.596929 | 1811.27322 | 2040.558327 | 1900.166667 | 2238.797269 | 2345.695052 | 0.912064688 | 0.304893409 | 1971.476159 | 2161.552996 |
| IL-12 p40 | 854.909089 | 930.4009771 | 789.648746 | 888.6666667 | 922.6267573 | 973.4204737 | 0.924676253 | 0.229907611 | 858.319604 | 928.2379659 |
| CCL24 | 165.795 | 172.3192 | 150.871 | 324.6666667 | 275.2158381 | 354.60772 | 0.512299851 | 0.01609653 | 162.9950667 | 318.1634083 |
| IL-3 | 205.5730189 | 250.0092249 | 190.2718191 | 208.6666667 | 228.5466461 | 263.4092354 | 0.921828829 | 0.490053255 | 215.2846876 | 233.5408494 |
| GMCSF | 44976.2865 | 55582.62453 | 46947.05326 | 50402.16667 | 45909.24547 | 59695.69493 | 0.945507978 | 0.616331867 | 49168.65476 | 52002.36902 |
| IFN-gamma | 571.6560776 | 365.3298609 | 535.4930601 | 457.6666667 | 461.8926062 | 436.256625 | 1.086046417 | 0.604144619 | 490.8263329 | 451.9386326 |
| IL-7 | 639.0359606 | 1570.874048 | 1045.874104 | 972.1666667 | 849.1476039 | 986.789885 | 1.159424271 | 0.637327852 | 1085.261371 | 936.0347185 |
| GCSF | 41.20043802 | 53.52060285 | 81.82102156 | 46.66666667 | 59.74318554 | 70.50772888 | 0.997877437 | 0.993330425 | 58.84735414 | 58.97252703 |
| IL-1 beta | 228.7482653 | 191.0182842 | 225.8701725 | 222.1666667 | 207.1979731 | 228.0757912 | 0.982045964 | 0.791654451 | 215.2122407 | 219.1468103 |
| IL-1 alpha | 263.0819636 | 204.7680523 | 207.6570615 | 250.1666667 | 196.771877 | 221.3910855 | 1.010739384 | 0.926961317 | 225.1690258 | 222.7765431 |

**by RayBio Human Cytokine Antibody Array**

**Table S8 Clinicopathologic characteristics of ccRCC patients divided by CCL24 expression in the training cohort (n=128).**

| **Characteristic** | **CCL24** | | **Sum**  **(128)** | **P***  **value** |
| --- | --- | --- | --- | --- |
|  | **Low expression (n=80)** | **High expression (n=48)** |  |  |
| **Age** |  |  |  | 0.258 |
| <60 | 47 | 33 | 80 |  |
| ≥60 | 33 | 15 | 48 |  |
| **Gender** |  |  |  | 0.695 |
| Male | 64 | 37 | 101 |  |
| Female | 16 | 11 | 27 |  |
| **WHO/ISUP grade** |  |  |  | 0.054 |
| I-II | 68 | 34 | 102 |  |
| III-IV | 12 | 14 | 26 |  |
| **TNM stage** |  |  |  | **0.019*** |
| I-II | 70 | 34 | 104 |  |
| III-IV | 10 | 14 | 24 |  |
| **SSIGN** |  |  |  | **0.005*** |
| 0-4 | 72 | 34 | 106 |  |
| ≥5 | 8 | 14 | 22 |  |
| **Gankyrin** |  |  |  | **<0.001*** |
| Low | 62 | 11 | 73 |  |
| High | 18 | 37 | 55 |  |
| *: Statistical significance was calculated by chi-square test or fisher's exact test for categorical/binary measures and ANOVA for continuous measures | | | | |

**Table S9 Clinicopathologic characteristics of ccRCC patients by CCL24 expression in the validation cohort (n=128)**

| **Characteristic** | **CCL24** | | **Sum**  **(128)** | **P***  **value** |
| --- | --- | --- | --- | --- |
|  | **Low expression (n=72)** | **High expression (n=56)** |  |  |
| **Age** |  |  |  | 0.789 |
| <60 | 39 | 29 | 68 |  |
| ≥60 | 33 | 27 | 60 |  |
| **Gender** |  |  |  | 0.294 |
| Male | 41 | 37 | 78 |  |
| Female | 31 | 19 | 50 |  |
| **WHO/ISUP grade** |  |  |  | **0.024*** |
| I-II | 62 | 39 | 101 |  |
| III-IV | 10 | 17 | 27 |  |
| **TNM stage** |  |  |  | **0.011*** |
| I-II | 65 | 41 | 106 |  |
| III-IV | 7 | 15 | 22 |  |
| **SSIGN** |  |  |  | **0.040*** |
| 0-4 | 63 | 41 | 104 |  |
| ≥5 | 9 | 15 | 24 |  |
| **Gankyrin** |  |  |  | **<0.001*** |
| Low | 66 | 27 | 93 |  |
| High | 6 | 29 | 35 |  |
| *: Statistical significance was calculated by chi-square test or fisher's exact test for categorical/binary measures and ANOVA for continuous measures | | | | |

**Table S10 Clinicopathologic characteristics of ccRCC patients by CCL24 expression in the combined cohort (n=256)**

| **Characteristic** | **CCL24** | | **Sum**  **(256)** | **P***  **value** |
| --- | --- | --- | --- | --- |
|  | **Low expression (n=152)** | **High expression (n=104)** |  |  |
| **Age** |  |  |  | 0.629 |
| <60 | 86 | 62 | 148 |  |
| ≥60 | 66 | 42 | 108 |  |
| **Gender** |  |  |  | 0.722 |
| Male | 105 | 74 | 179 |  |
| Female | 47 | 30 | 77 |  |
| **WHO/ISUP grade** |  |  |  | **0.008*** |
| I-II | 129 | 74 | 203 |  |
| III-IV | 23 | 30 | 53 |  |
| **TNM stage** |  |  |  | **<0.001*** |
| I-II | 135 | 75 | 210 |  |
| III-IV | 17 | 29 | 46 |  |
| **SSIGN** |  |  |  | **<0.001*** |
| 0-4 | 136 | 74 | 210 |  |
| ≥5 | 16 | 30 | 46 |  |
| **Gankyrin** |  |  |  | **<0.001*** |
| Low | 128 | 38 | 166 |  |
| High | 24 | 66 | 90 |  |
| *: Statistical significance was calculated by chi-square test or fisher's exact test for categorical/binary measures and ANOVA for continuous measures | | | | |

**Table S11 Gankyrin-interacting significantly expressed proteins identified by NanoLC-ESI-MS（MS）**

| **General Name** | **Link** | **Protein Mass** | **Gankyrin** | **IgG** | **Sequence Header** |
| --- | --- | --- | --- | --- | --- |
| ACTB | [P60709](http://www.uniprot.org/uniprot/P60709) | 42051.85 | 78.4% | #N/A | >sp\|P60709\|ACTB_HUMAN Actin, cytoplasmic 1 OS=Homo sapiens GN=ACTB PE=1 SV=1 |
| MYH9 | [P35579](http://www.uniprot.org/uniprot/P35579) | 227645.05 | 4.1% | #N/A | >sp\|P35579\|MYH9_HUMAN Myosin-9 OS=Homo sapiens GN=MYH9 PE=1 SV=4 |
| PSMD10 | [O75832](https://www.uniprot.org/uniprot/O75832) | 24427.83 | 2.1% | #N/A | >sp\|O75832\|PSMD10_HUMAN 26S proteasome non-ATPase regulatory subunit 10 OS=Homo sapiens GN=PSMD10 PE=1 SV=1 |
| MYO1C | [O00159](http://www.uniprot.org/uniprot/O00159) | 122461.38 | 1.9% | #N/A | >sp\|O00159\|MYO1C_HUMAN Unconventional myosin-Ic OS=Homo sapiens GN=MYO1C PE=1 SV=4 |
| TUBA1B | [P68363](http://www.uniprot.org/uniprot/P68363) | 50803.87 | 1.8% | #N/A | >sp\|P68363\|TBA1B_HUMAN Tubulin alpha-1B chain OS=Homo sapiens GN=TUBA1B PE=1 SV=1 |
| ALB | [P02768](http://www.uniprot.org/uniprot/P02768) | 71317.36 | 1.4% | #N/A | >sp\|P02768\|ALBU_HUMAN Serum albumin OS=Homo sapiens GN=ALB PE=1 SV=2 |
| TUBB | [P07437](http://www.uniprot.org/uniprot/P07437) | 50095.16 | 1.1% | #N/A | >sp\|P07437\|TBB5_HUMAN Tubulin beta chain OS=Homo sapiens GN=TUBB PE=1 SV=2 |
| MYL6 | [P60660](http://www.uniprot.org/uniprot/P60660) | 17090.19 | 0.9% | #N/A | >sp\|P60660\|MYL6_HUMAN Myosin light polypeptide 6 OS=Homo sapiens GN=MYL6 PE=1 SV=2 |
| NONO | [Q15233](http://www.uniprot.org/uniprot/Q15233) | 54311.35 | 0.8% | #N/A | >sp\|Q15233\|NONO_HUMAN Non-POU domain-containing octamer-binding protein OS=Homo sapiens GN=NONO PE=1 SV=4 |
| MYL12B | [O14950](http://www.uniprot.org/uniprot/O14950) | 19823.54 | 0.8% | #N/A | >sp\|O14950\|ML12B_HUMAN Myosin regulatory light chain 12B OS=Homo sapiens GN=MYL12B PE=1 SV=2 |
| CLTC | [Q00610](http://www.uniprot.org/uniprot/Q00610) | 193259.28 | 0.7% | #N/A | >sp\|Q00610\|CLH1_HUMAN Clathrin heavy chain 1 OS=Homo sapiens GN=CLTC PE=1 SV=5 |
| EEF1A1P5 | [Q5VTE0](http://www.uniprot.org/uniprot/Q5VTE0) | 50495.29 | 0.6% | #N/A | >sp\|Q5VTE0\|EF1A3_HUMAN Putative elongation factor 1-alpha-like 3 OS=Homo sapiens GN=EEF1A1P5 PE=5 SV=1 |
| C10orf67 | [Q8IYJ2](http://www.uniprot.org/uniprot/Q8IYJ2) | 63843.98 | 0.5% | #N/A | >sp\|Q8IYJ2\|CJ067_HUMAN Uncharacterized protein C10orf67, mitochondrial OS=Homo sapiens GN=C10orf67 PE=2 SV=3 |
| SFPQ | [P23246](http://www.uniprot.org/uniprot/P23246) | 76215.77 | 0.4% | #N/A | >sp\|P23246\|SFPQ_HUMAN Splicing factor, proline- and glutamine-rich OS=Homo sapiens GN=SFPQ PE=1 SV=2 |
| HSPA9 | [P38646](http://www.uniprot.org/uniprot/P38646) | 73919.87 | 0.4% | #N/A | >sp\|P38646\|GRP75_HUMAN Stress-70 protein, mitochondrial OS=Homo sapiens GN=HSPA9 PE=1 SV=2 |
| **STAT3** | P40763 | 88067.8 | 0.3% | #N/A | >sp\|P40763\|STAT3_Signal transducer and activator of transcription 3 OS=Homo sapiens **GN=STAT3** PE=1 SV=1 |
| PLEC | [Q15149](http://www.uniprot.org/uniprot/Q15149) | 533461.94 | 0.3% | #N/A | >sp\|Q15149\|PLEC_HUMAN Plectin OS=Homo sapiens GN=PLEC PE=1 SV=3 |
| MYO6 | [A0A0A0MRM8](http://www.uniprot.org/uniprot/A0A0A0MRM8) | 146292.39 | 0.3% | #N/A | >tr\|A0A0A0MRM8\|A0A0A0MRM8_HUMAN Unconventional myosin-VI OS=Homo sapiens GN=MYO6 PE=1 SV=1 |
| MYO6 | [A0A0D9SGC1](http://www.uniprot.org/uniprot/A0A0D9SGC1) | 150909.48 | 0.2% | #N/A | >tr\|A0A0D9SGC1\|A0A0D9SGC1_HUMAN Unconventional myosin-VI OS=Homo sapiens GN=MYO6 PE=1 SV=1 |
| NPM1 | [P06748](http://www.uniprot.org/uniprot/P06748) | 32725.92 | 0.2% | #N/A | >sp\|P06748\|NPM_HUMAN Nucleophosmin OS=Homo sapiens GN=NPM1 PE=1 SV=2 |
| RPL29 | [P47914](http://www.uniprot.org/uniprot/P47914) | 17798.07 | 0.2% | #N/A | >sp\|P47914\|RL29_HUMAN 60S ribosomal protein L29 OS=Homo sapiens GN=RPL29 PE=1 SV=2 |
| TRAP1 | [Q12931](http://www.uniprot.org/uniprot/Q12931) | 80344.86 | 0.2% | #N/A | >sp\|Q12931\|TRAP1_HUMAN Heat shock protein 75 kDa, mitochondrial OS=Homo sapiens GN=TRAP1 PE=1 SV=3 |
| DKFZp686J1372 | [Q5HYB6](http://www.uniprot.org/uniprot/Q5HYB6) | 27386.88 | 0.2% | #N/A | >tr\|Q5HYB6\|Q5HYB6_HUMAN Epididymis luminal protein 189 OS=Homo sapiens GN=DKFZp686J1372 PE=1 SV=1 |
| RPL14 | [Q6IPH7](http://www.uniprot.org/uniprot/Q6IPH7) | 23886.24 | 0.2% | #N/A | >tr\|Q6IPH7\|Q6IPH7_HUMAN RPL14 protein OS=Homo sapiens GN=RPL14 PE=1 SV=1 |
| RPL7 | [P18124](http://www.uniprot.org/uniprot/P18124) | 29264.21 | 0.2% | #N/A | >sp\|P18124\|RL7_HUMAN 60S ribosomal protein L7 OS=Homo sapiens GN=RPL7 PE=1 SV=1 |
| CLINT1 | [A0A0S2Z5H3](http://www.uniprot.org/uniprot/A0A0S2Z5H3) | 70306.91 | 0.1% | #N/A | >tr\|A0A0S2Z5H3\|A0A0S2Z5H3_HUMAN Clathrin interactor 1 isoform 2 (Fragment) OS=Homo sapiens GN=CLINT1 PE=2 SV=1 |
| HSPA8 | [P11142](http://www.uniprot.org/uniprot/P11142) | 71082.38 | 0.1% | #N/A | >sp\|P11142\|HSP7C_HUMAN Heat shock cognate 71 kDa protein OS=Homo sapiens GN=HSPA8 PE=1 SV=1 |
| HNRNPH1 | [P31943](http://www.uniprot.org/uniprot/P31943) | 49483.49 | 0.1% | #N/A | >sp\|P31943\|HNRH1_HUMAN Heterogeneous nuclear ribonucleoprotein H OS=Homo sapiens GN=HNRNPH1 PE=1 SV=4 |
| RPL6 | [Q02878](http://www.uniprot.org/uniprot/Q02878) | 32764.64 | 0.1% | #N/A | >sp\|Q02878\|RL6_HUMAN 60S ribosomal protein L6 OS=Homo sapiens GN=RPL6 PE=1 SV=3 |
| ACTN4 | [O43707](http://www.uniprot.org/uniprot/O43707) | 105244.61 | 0.1% | #N/A | >sp\|O43707\|ACTN4_HUMAN Alpha-actinin-4 OS=Homo sapiens GN=ACTN4 PE=1 SV=2 |
| RPS2 | [P15880](http://www.uniprot.org/uniprot/P15880) | 31589.68 | 0.1% | #N/A | >sp\|P15880\|RS2_HUMAN 40S ribosomal protein S2 OS=Homo sapiens GN=RPS2 PE=1 SV=2 |
| XPNPEP3 | [Q9NQH7](http://www.uniprot.org/uniprot/Q9NQH7) | 57624.14 | 0.1% | #N/A | >sp\|Q9NQH7\|XPP3_HUMAN Probable Xaa-Pro aminopeptidase 3 OS=Homo sapiens GN=XPNPEP3 PE=1 SV=1 |
| RPL18 | [Q07020](http://www.uniprot.org/uniprot/Q07020) | 21735.1 | 0.1% | #N/A | >sp\|Q07020\|RL18_HUMAN 60S ribosomal protein L18 OS=Homo sapiens GN=RPL18 PE=1 SV=2 |
| ATP5A1 | [P25705](http://www.uniprot.org/uniprot/P25705) | 59827.72 | 0.1% | #N/A | >sp\|P25705\|ATPA_HUMAN ATP synthase subunit alpha, mitochondrial OS=Homo sapiens GN=ATP5A1 PE=1 SV=1 |
| MYL3 | [P08590](http://www.uniprot.org/uniprot/P08590) | 22088.98 | 0.1% | #N/A | >sp\|P08590\|MYL3_HUMAN Myosin light chain 3 OS=Homo sapiens GN=MYL3 PE=1 SV=3 |
| PKM | [P14618](http://www.uniprot.org/uniprot/P14618) | 58470.32 | 0.1% | #N/A | >sp\|P14618\|KPYM_HUMAN Pyruvate kinase PKM OS=Homo sapiens GN=PKM PE=1 SV=4 |
| RPL4 | [P36578](http://www.uniprot.org/uniprot/P36578) | 47952.58 | 0.1% | #N/A | >sp\|P36578\|RL4_HUMAN 60S ribosomal protein L4 OS=Homo sapiens GN=RPL4 PE=1 SV=5 |
| NAA50 | [Q9GZZ1](http://www.uniprot.org/uniprot/Q9GZZ1) | 19614.12 | 0.1% | #N/A | >sp\|Q9GZZ1\|NAA50_HUMAN N-alpha-acetyltransferase 50 OS=Homo sapiens GN=NAA50 PE=1 SV=1 |
| MYO1E | [Q12965](http://www.uniprot.org/uniprot/Q12965) | 127552.16 | 0.1% | #N/A | >sp\|Q12965\|MYO1E_HUMAN Unconventional myosin-Ie OS=Homo sapiens GN=MYO1E PE=1 SV=2 |

**Table S12 Clinicopathologic characteristics of ccRCC patients by gankyrin and STAT3 expression in the training cohort (n=128).**

|  | **Gankyrin/STAT3 expression** | | | | |  |
| --- | --- | --- | --- | --- | --- | --- |
| **Characteristic** | **Gankyrin^high^**  **STAT3^low^**  **(n=2)** | **Gankyrin^low^**  **STAT3^low^**  **(n=57)** | **Gankyrin^high^**  **STAT3^high^**  **(n=53)** | **Gankyrin^low^**  **STAT3^high^**  **(n=16)** | **sum**  **(128)** | ***P****  **value** |
| **Age** |  |  |  |  |  | 0.986 |
| <60 | 1 | 36 | 33 | 10 | 80 |  |
| ≥60 | 1 | 21 | 20 | 6 | 48 |  |
| **Gender** |  |  |  |  |  | 0.384 |
| Male | 1 | 47 | 39 | 14 | 101 |  |
| Female | 1 | 10 | 14 | 2 | 27 |  |
| **WHO/ISUP grade** |  |  |  |  |  | 0.293 |
| 1-2 | 2 | 46 | 39 | 15 | 102 |  |
| 3-4 | 0 | 11 | 14 | 1 | 26 |  |
| **TNM stage** |  |  |  |  |  | **0.011*** |
| 1-2 | 1 | 50 | 37 | 16 | 104 |  |
| 3-4 | 1 | 7 | 16 | 0 | 24 |  |
| **SSIGN** |  |  |  |  |  | 0.094 |
| 0-4 | 1 | 52 | 40 | 13 | 106 |  |
| ≥5 | 1 | 5 | 13 | 3 | 22 |  |

*: Statistical significance was calculated by chi-square test or fisher's exact test

for categorical/binary measures and ANOVA for continuous measures

**Table S13 Clinicopathologic characteristics of ccRCC patients by gankyrin and STAT3 expression in the validation cohort (n=128).**

|  | **Gankyrin/STAT3 expression** | | | | |  |
| --- | --- | --- | --- | --- | --- | --- |
| **Characteristic** | **Gankyrin^high^**  **STAT3^low^**  **(n=5)** | **Gankyrin^low^**  **STAT3^low^**  **(n=65)** | **Gankyrin^high^**  **STAT3^high^**  **(n=30)** | **Gankyrin^low^**  **STAT3^high^**  **(n=28)** | **sum (128)** | ***P** value** |
| **Age** |  |  |  |  |  | 0.897 |
| <60 | 2 | 36 | 15 | 15 | 68 |  |
| ≥60 | 3 | 29 | 15 | 13 | 60 |  |
| **Gender** |  |  |  |  |  | 0.347 |
| Male | 4 | 35 | 21 | 18 | 78 |  |
| Female | 1 | 30 | 9 | 10 | 50 |  |
| **WHO/ISUP grade** |  |  |  |  |  | 0.093 |
| 1-2 | 4 | 56 | 19 | 22 | 101 |  |
| 3-4 | 1 | 9 | 11 | 6 | 27 |  |
| **TNM stage** |  |  |  |  |  | \| **0.004*** \| \| --- \| |
| 1-2 | 5 | 60 | 19 | 22 | 106 |  |
| 3-4 | 0 | 5 | 11 | 6 | 22 |  |
| **SSIGN** |  |  |  |  |  | **0.016*** |
| 0-4 | 5 | 58 | 19 | 22 | 104 |  |
| ≥5 | 0 | 7 | 11 | 6 | 24 |  |

*: Statistical significance was calculated by chi-square test or fisher's exact test for

categorical/binary measures and ANOVA for continuous measures

**Table S14 Clinicopathologic characteristics of ccRCC patients by gankyrin and STAT3 expression in the combined cohort (n=256)**

|  | **Gankyrin/STAT3 expression** | | | | |  |
| --- | --- | --- | --- | --- | --- | --- |
| **Characteristic** | **Gankyrin^high^**  **STAT3^low^**  **(n=7)** | **Gankyrin^low^**  **STAT3^low^**  **(n=122)** | **Gankyrin^high^**  **STAT3^high^**  **(n=83)** | **Gankyrin^low^**  **STAT3^high^**  **(n=44)** | **sum (256)** | ***P** value** |
| **Age** |  |  |  |  |  | 0.866 |
| <60 | 3 | 72 | 48 | 25 | 148 |  |
| ≥60 | 4 | 50 | 35 | 19 | 108 |  |
| **Gender** |  |  |  |  |  | 0.845 |
| Male | 5 | 82 | 60 | 32 | 179 |  |
| Female | 2 | 40 | 23 | 12 | 77 |  |
| **WHO/ISUP grade** |  |  |  |  |  |  |
| 1-2 | 6 | 102 | 58 | 37 | 203 | 0.084 |
| 3-4 | 1 | 20 | 25 | 7 | 53 |  |
| **TNM stage** |  |  |  |  |  | **<0.001*** |
| 1-2 | 6 | 110 | 56 | 38 | 210 |  |
| 3-4 | 1 | 12 | 27 | 6 | 46 |  |
| **SSIGN** |  |  |  |  |  | **0.006*** |
| 0-4 | 6 | 110 | 59 | 35 | 210 |  |
| ≥5 | 1 | 12 | 24 | 9 | 46 |  |

*: Statistical significance was calculated by chi-square test or fisher's exact test

for categorical/binary measures and ANOVA for continuous measures

**Table S15 Clinicopathologic characteristics of ccRCC patients by gankyrin and CCL24 expression in the training cohort (n=128).**

|  | **Gankyrin/CCL24 expression** | | | | |  |
| --- | --- | --- | --- | --- | --- | --- |
| **Characteristic** | **Gankyrin^high^**  **CCL24^low^**  **(n=18)** | **Gankyrin^low^**  **CCL24^low^**  **(n=62)** | **Gankyrin^high^**  **CCL24^high^**  **(n=37)** | **Gankyrin^low^**  **CCL24^high^**  **(n=11)** | **sum**  **(128)** | ***P****  **value** |
| **Age** |  |  |  |  |  | 0.490 |
| <60 | 10 | 37 | 24 | 9 | 80 |  |
| ≥60 | 8 | 25 | 13 | 2 | 48 |  |
| **Gender** |  |  |  |  |  | 0.451 |
| Male | 13 | 51 | 27 | 10 | 101 |  |
| Female | 5 | 11 | 10 | 1 | 27 |  |
| **WHO/ISUP grade** |  |  |  |  |  | **0.036*** |
| 1-2 | 17 | 51 | 24 | 10 | 102 |  |
| 3-4 | 1 | 11 | 13 | 1 | 26 |  |
| **TNM stage** |  |  |  |  |  | **0.003*** |
| 1-2 | 15 | 55 | 23 | 11 | 104 |  |
| 3-4 | 3 | 7 | 14 | 0 | 24 |  |
| **SSIGN** |  |  |  |  |  | **0.030*** |
| 0-4 | 16 | 56 | 25 | 9 | 106 |  |
| ≥5 | 2 | 6 | 12 | 2 | 22 |  |

*: Statistical significance was calculated by chi-square test or fisher's exact test

for categorical/binary measures and ANOVA for continuous measures

**Table S16 Clinicopathologic characteristics of ccRCC patients by gankyrin and CCL24 expression in the validation cohort (n=128).**

|  | **Gankyrin/CCL24 expression** | | | | |  |
| --- | --- | --- | --- | --- | --- | --- |
| **Characteristic** | **Gankyrin^high^**  **CCL24^low^**  **(n=6)** | **Gankyrin^low^**  **CCL24^low^**  **(n=66)** | **Gankyrin^high^**  **CCL24^high^**  **(n=29)** | **Gankyrin^low^**  **CCL24^high^**  **(n=27)** | **sum (128)** | ***P** value** |
| **Age** |  |  |  |  |  | 0.680 |
| <60 | 2 | 37 | 14 | 15 | 68 |  |
| ≥60 | 4 | 29 | 15 | 12 | 60 |  |
| **Gender** |  |  |  |  |  | 0.665 |
| Male | 4 | 37 | 20 | 17 | 78 |  |
| Female | 2 | 29 | 9 | 10 | 50 |  |
| **WHO/ISUP grade** |  |  |  |  |  | 0.075 |
| 1-2 | 4 | 58 | 21 | 18 | 101 |  |
| 3-4 | 2 | 8 | 8 | 9 | 27 |  |
| **TNM stage** |  |  |  |  |  | **0.021*** |
| 1-2 | 4 | 61 | 20 | 21 | 106 |  |
| 3-4 | 2 | 5 | 9 | 6 | 22 |  |
| **SSIGN** |  |  |  |  |  | **0.012*** |
| 0-4 | 3 | 60 | 20 | 21 | 104 |  |
| ≥5 | 3 | 6 | 9 | 6 | 24 |  |

*: Statistical significance was calculated by chi-square test or fisher's exact test for

categorical/binary measures and ANOVA for continuous measures

**Table S17 Clinicopathologic characteristics of ccRCC patients by gankyrin and CCL24 expression in the combined cohort (n=256)**

|  | **Gankyrin/CCL24 expression** | | | | |  |
| --- | --- | --- | --- | --- | --- | --- |
| **Characteristic** | **Gankyrin^high^**  **CCL24^low^**  **(n=24)** | **Gankyrin^low^**  **CCL24^low^**  **(n=128)** | **Gankyrin^high^**  **CCL24^high^**  **(n=66)** | **Gankyrin^low^**  **CCL24^high^**  **(n=38)** | **sum (256)** | ***P** value** |
| **Age** |  |  |  |  |  | 0.790 |
| <60 | 12 | 74 | 38 | 24 | 148 |  |
| ≥60 | 12 | 54 | 28 | 14 | 108 |  |
| **Gender** |  |  |  |  |  | 0.983 |
| Male | 17 | 88 | 47 | 27 | 179 |  |
| Female | 7 | 40 | 19 | 11 | 77 |  |
| **WHO/ISUP grade** |  |  |  |  |  | **0.025*** |
| 1-2 | 21 | 109 | 45 | 28 | 203 |  |
| 3-4 | 3 | 19 | 21 | 10 | 53 |  |
| **TNM stage** |  |  |  |  |  | **<0.001*** |
| 1-2 | 19 | 116 | 43 | 32 | 210 |  |
| 3-4 | 5 | 12 | 23 | 6 | 46 |  |
| **SSIGN** |  |  |  |  |  | **0.002*** |
| 0-4 | 19 | 116 | 45 | 30 | 210 |  |
| ≥5 | 5 | 12 | 21 | 8 | 46 |  |

*: Statistical significance was calculated by chi-square test or fisher's exact test

for categorical/binary measures and ANOVA for continuous measures

**Table S18 Univariate and multivariate cox regression analysis of gankyrin, STAT3 and clinical characteristics**

**with overall survival and progression-free survival in the training cohort (n=128).**

|  | **Overall survival** | | | | **Progression-free survival** | | | |
| --- | --- | --- | --- | --- | --- | --- | --- | --- |
| **Characteristics** | **Univariate** | | **Multivariate** | | **Univariate** | | **Multivariate** | |
|  | **HR (95% CI)** | ***P* Value** | **HR (95% CI)** | ***P* Value** | **HR (95% CI)** | ***P* Value** | **HR (95% CI)** | ***P* Value** |
| **Age**  **(<60y vs ≥60y)** | 1.011  (0.462-2.213) | 0.978 |  |  | 1.232 (0.693-2.19) | 0.476 |  |  |
| **Gender**  **(Male vs Female)** | 2.037  (0.914-4.538) | 0.082 |  |  | 1.324 (0.688-2.546) | 0.401 |  |  |
| **WHO/ISUP grade**  **(1-2 vs 3-4)** | 2.195  (0.977-4.93) | **0.035** | 0.992 (0.289-3.399) | 0.099 | 1.421 (0.737-2.739) | **0.029** | 0.850  (0.369-1.957) | 0.070 |
| **TNM stage**  **(1-2 vs 3-4)** | 3.448  (1.582-7.513) | **0.002** | 2.356  (0.566-9.8) | **0.024** | 2.463 (1.346-4.507) | **0.003** | 2.360  (0.91-6.12) | **0.027** |
| **SSIGN**  **(1-4 vs ≥5)** | 3.536  (1.601-7.811) | **0.002** | 1.420  (0.433-4.658) | **0.025** | 2.048 (1.079-3.887) | **0.028** | 1.351 (0.604-3.025) | 0.064 |
| **Gankyrin expression**  **(Low vs High)** | 1.025  (1.015-1.035) | **<0.001** | 1.022 (1.011-1.034) | **<0.001** | 1.011 (1.005-1.018) | **<0.001** | 1.010  (1.003-1.017) | **0.007** |
| **STAT3 expression**  **(Low vs High)** | 1.044  (1.017-1.072) | **0.001** | 1.008 (0.978-1.039) | **0.026** | 1.025 (1.006-1.045) | **0.002** | 1.005 (0.982-1.029) | **0.029** |

**Table S19 Univariate and multivariate cox regression analysis of gankyrin, STAT3 and clinical characteristics**

**with overall survival and progression-free survival in the validation cohort (n=128)**

|  | **Overall survival** | | | | **Progression-free survival** | | | |
| --- | --- | --- | --- | --- | --- | --- | --- | --- |
| **Characteristics** | **Univariate** | | **Multivariate** | | **Univariate** | | **Multivariate** | |
|  | **HR (95% CI)** | ***P* Value** | **HR (95% CI)** | ***P* Value** | **HR (95% CI)** | ***P* Value** | **HR (95% CI)** | ***P* Value** |
| **Age**  **(<60y vs ≥60y)** | 1.031 (0.408-2.607) | 0.948 |  |  | 0.891  (0.498-1.593) | 0.697 |  |  |
| **Gender**  **(Male vs Female)** | 1.247 (0.492-3.162) | 0.641 |  |  | 0.811  (0.442-1.488) | 0.499 |  |  |
| **WHO/ISUP grade**  **(1-2 vs 3-4)** | 3.602 (1.407-9.219) | **0.008** | 1.630 (0.386-6.878) | 0.056 | 1.849  (0.971-3.523) | **0.026** | 0.782 (0.275-2.222) | 0.064 |
| **TNM stage**  **(1-2 vs 3-4)** | 5.573 (2.153-14.428) | **<0.001** | 2.508 (0.529-11.897) | **0.025** | 3.118  (1.622-5.994) | **0.001** | 3.811  (1.24-11.71) | **0.020** |
| **SSIGN**  **(1-4 vs ≥5)** | 2.108 (0.789-5.632) | **0.003** | 0.735 (0.236-2.29) | **0.045** | 1.536  (0.78-3.026) | **0.005** | 0.758 (0.338-1.702) | 0.050 |
| **Gankyrin expression**  **(Low vs High)** | 1.019 (1.007-1.031) | **0.001** | 1.010  (0.991-1.03) | **0.035** | 1.010  (1.004-1.016) | **0.001** | 1.003 (0.991-1.016) | **0.036** |
| **STAT3 expression**  **(Low vs High)** | 1.047 (1.016-1.078) | **0.003** | 1.022 (0.969-1.078) | **0.043** | 1.026  (1.01-1.041) | **0.001** | 1.015 (0.984-1.047) | **0.034** |

**Table S20 Univariate and multivariate cox regression analysis of gankyrin, STAT3 and clinical characteristics**

**with overall survival and progression-free survival in the combined cohort (n=256)**

|  | **Overall survival** | | | | **Progression-free survival** | | | |
| --- | --- | --- | --- | --- | --- | --- | --- | --- |
| **Characteristics** | **Univariate** | | **Multivariate** | | **Univariate** | | **Multivariate** | |
|  | **HR (95% CI)** | ***P* Value** | **HR (95% CI)** | ***P* Value** | **HR (95% CI)** | ***P* Value** | **HR (95% CI)** | ***P* Value** |
| **Age**  **(<60y vs ≥60y)** | 0.975 (0.539-1.763) | 0.933 |  |  | 1.019  (0.677-1.532) | 0.930 |  |  |
| **Gender**  **(Male vs Female)** | 1.467 (0.803-2.682) | 0.213 |  |  | 1.029  (0.661-1.601) | 0.900 |  |  |
| **WHO/ISUP grade**  **(1-2 vs 3-4)** | 2.724 (1.486-4.991) | **0.001** | 1.218 (0.512-2.896) | 0.056 | 1.626  (1.029-2.572) | **0.037** | 0.880  (0.484-1.599) | 0.097 |
| **TNM stage**  **(1-2 vs 3-4)** | 4.388 (2.411-7.985) | **<0.001** | 2.214  (0.838-5.85) | **0.019** | 2.797  (1.801-4.342) | **<0.001** | 2.700  (1.423-5.125) | **0.002** |
| **SSIGN**  **(1-4 vs ≥5)** | 2.804 (1.519-5.177) | **0.001** | 1.152 (0.538-2.467) | **0.016** | 1.787  (1.123-2.842) | **0.014** | 1.027 (0.581-1.816) | **0.026** |
| **Gankyrin expression**  **(Low vs High)** | 1.021 (1.015-1.028) | **<0.001** | 1.018  (1.01-1.027) | **<0.001** | 1.010  (1.006-1.014) | **<0.001** | 1.007 (1.001-1.012) | **0.022** |
| **STAT3 expression**  **(Low vs High)** | 1.047 (1.027-1.068) | **<0.001** | 1.007 (0.983-1.032) | **0.037** | 1.025 (1.013-1.037) | **<0.001** | 1.007  (0.99-1.024) | **0.042** |

**Table S21 Univariate and multivariate cox regression analysis of gankyrin, CCL24 and clinical characteristics**

**with overall survival and progression-free survival in the training cohort (n=128).**

|  | **Overall survival** | | | | **Progression-free survival** | | | |
| --- | --- | --- | --- | --- | --- | --- | --- | --- |
| **Characteristics** | **Univariate** | | **Multivariate** | | **Univariate** | | **Multivariate** | |
|  | **HR (95% CI)** | ***P* Value** | **HR (95% CI)** | ***P* Value** | **HR (95% CI)** | ***P* Value** | **HR (95% CI)** | ***P* Value** |
| **Age**  **(<60y vs ≥60y)** | 1.011  (0.462-2.213) | 0.978 |  |  | 1.232 (0.693-2.19) | 0.476 |  |  |
| **Gender**  **(Male vs Female)** | 2.037  (0.914-4.538) | 0.082 |  |  | 1.324 (0.688-2.546) | 0.401 |  |  |
| **WHO/ISUP grade**  **(1-2 vs 3-4)** | 2.195  (0.977-4.93) | **0.035** | 0.911 (0.261-3.185) | 0.088 | 1.421 (0.737-2.739) | **0.029** | 0.840  (0.361-1.956) | 0.069 |
| **TNM stage**  **(1-2 vs 3-4)** | 3.448  (1.582-7.513) | **0.002** | 2.837 (0.654-12.306) | **0.016** | 2.463 (1.346-4.507) | **0.003** | 2.528 (0.968-6.601) | **0.038** |
| **SSIGN**  **(1-4 vs ≥5)** | 3.536  (1.601-7.811) | **0.002** | 1.415  (0.426-4.7) | **0.017** | 2.048 (1.079-3.887) | **0.028** | 1.332 (0.592-2.994) | **0.049** |
| **Gankyrin expression**  **(Low vs High)** | 1.025  (1.015-1.035) | **<0.001** | 1.022  (1.01-1.035) | **<0.001** | 1.011 (1.005-1.018) | **<0.001** | 1.010  (1.003-1.017) | **0.008** |
| **CCL24 expression**  **(Low vs High)** | 1.015  (1.007-1.023) | **<0.001** | 1.005 (0.996-1.014) | **0.009** | 1.009 (1.003-1.014) | **0.004** | 1.003  (0.996-1.01) | **0.023** |

**Table S22 Univariate and multivariate cox regression analysis of gankyrin, CCL24 and clinical characteristics**

**with overall survival and progression-free survival in the validation cohort (n=128)**

|  | **Overall survival** | | | | **Progression-free survival** | | | |
| --- | --- | --- | --- | --- | --- | --- | --- | --- |
| **Characteristics** | **Univariate** | | **Multivariate** | | **Univariate** | | **Multivariate** | |
|  | **HR (95% CI)** | ***P* Value** | **HR (95% CI)** | ***P* Value** | **HR (95% CI)** | ***P* Value** | **HR (95% CI)** | ***P* Value** |
| **Age**  **(<60y vs ≥60y)** | 1.031 (0.408-2.607) | 0.948 |  |  | 0.891  (0.498-1.593) | 0.697 |  |  |
| **Gender**  **(Male vs Female)** | 1.247 (0.492-3.162) | 0.641 |  |  | 0.811  (0.442-1.488) | 0.499 |  |  |
| **WHO/ISUP grade**  **(1-2 vs 3-4)** | 3.602 (1.407-9.219) | **0.008** | 1.669 (0.395-7.052) | 0.054 | 1.849  (0.971-3.523) | **0.026** | 0.762  (0.27-2.147) | 0.067 |
| **TNM stage**  **(1-2 vs 3-4)** | 5.573 (2.153-14.428) | **<0.001** | 2.460 (0.518-11.687) | **0.026** | 3.118  (1.622-5.994) | **0.001** | 3.813 (1.251-11.621) | **0.019** |
| **SSIGN (1-4 vs ≥5)** | 2.108 (0.789-5.632) | **0.003** | 0.720  (0.23-2.255) | **0.035** | 1.536  (0.78-3.026) | **0.005** | 0.780  (0.349-1.744) | **0.046** |
| **Gankyrin expression**  **(Low vs High)** | 1.019 (1.007-1.031) | **0.001** | 1.010 (0.991-1.029) | **0.023** | 1.010  (1.004-1.016) | **0.001** | 1.005  (0.992-1.017) | **0.025** |
| **CCL24 expression**  **(Low vs High)** | 1.016 (1.006-1.026) | **0.002** | 1.008 (0.991-1.026) | **0.037** | 1.008  (1.003-1.013) | **0.002** | 1.004  (0.993-1.014) | **0.031** |

**Table S23 Univariate and multivariate cox regression analysis of gankyrin, CCL24 and clinical characteristics**

**with overall survival and progression-free survival in the combined cohort (n=256)**

|  | **Overall survival** | | | | **Progression-free survival** | | | |
| --- | --- | --- | --- | --- | --- | --- | --- | --- |
| **Characteristics** | **Univariate** | | **Multivariate** | | **Univariate** | | **Multivariate** | |
|  | **HR (95% CI)** | ***P* Value** | **HR (95% CI)** | ***P* Value** | **HR (95% CI)** | ***P* Value** | **HR (95% CI)** | ***P* Value** |
| **Age**  **(<60y vs ≥60y)** | 0.975 (0.539-1.763) | 0.933 |  |  | 1.019  (0.677-1.532) | 0.930 |  |  |
| **Gender**  **(Male vs Female)** | 1.467 (0.803-2.682) | 0.213 |  |  | 1.029  (0.661-1.601) | 0.900 |  |  |
| **WHO/ISUP grade**  **(1-2 vs 3-4)** | 2.724 (1.486-4.991) | **0.001** | 1.183 (0.492-2.843) | 0.051 | 1.626  (1.029-2.572) | **0.037** | 0.868  (0.475-1.585) | 0.094 |
| **TNM stage**  **(1-2 vs 3-4)** | 4.388 (2.411-7.985) | **<0.001** | 2.403 (0.903-6.398) | **0.018** | 2.797  (1.801-4.342) | **<0.001** | 2.780  (1.465-5.275) | **0.002** |
| **SSIGN**  **(1-4 vs ≥5)** | 2.804 (1.519-5.177) | **0.001** | 1.107 (0.513-2.389) | **0.015** | 1.787  (1.123-2.842) | **0.014** | 1.012  (0.57-1.795) | **0.021** |
| **Gankyrin expression**  **(Low vs High)** | 1.021 (1.015-1.028) | **<0.001** | 1.018 (1.009-1.027) | **<0.001** | 1.010  (1.006-1.014) | **<0.001** | 1.007  (1.001-1.012) | **0.016** |
| **CCL24 expression**  **(Low vs High)** | 1.016 (1.009-1.022) | **<0.001** | 1.004 (0.997-1.012) | **0.024** | 1.008  (1.005-1.012) | **<0.001** | 1.003  (0.997-1.008) | **0.035** |

**Table S24 C-index analysis of the prognostic accuracy of gankyrin, CCL24 and other variables for OS and PFS**

**in the training, validation, and combined cohorts.**

|  | **Overall survival** | | | **Progression-free survival** | | |
| --- | --- | --- | --- | --- | --- | --- |
| 1. **index**   **(95% CI)** | **Training Cohort**  **(n=128)** | **Validation Cohort**  **(n=128)** | **Combine Cohort**  **(n=256)** | **Training Cohort**  **(n=128)** | **Validation Cohort**  **(n=128)** | **Combined Cohort**  **(n=256)** |
| **TNM stage** | 0.7225（0.6433-0.7617） | 0.7138（0.6344-0.7633） | 0.7334（0.6654-0.7815） | 0.6823（0.6113-0.7209） | 0.6674（0.6011-0.7069） | 0.6787（0.6141-0.7133） |
| **SSIGN** | 0.7159（0.6367-0.7551） | 0.7013（0.6218-0.7408） | 0.7205（0.6513-0.7697） | 0.6837（0.6149-0.7252） | 0.6637（0.6043-0.7188） | 0.6704（0.6089-0.7206） |
| **Gankyrin** | 0.7519（0.6330-0.8608） | 0.7617（0.6437-0.8834） | 0.7942（0.6880-0.9061） | 0.7387（0.6302-0.8359） | 0.7138（0.6179-0.8271） | 0.7277（0.6241-0.8305） |
| **CCL24** | 0.7321（0.6342-0.8501） | 0.7336（0.6401-0.8671） | 0.7756（0.6832-0.9069） | 0.7233（0.6258-0.8216） | 0.7118（0.6235-0.8301） | 0.7169（0.6222-0.8237） |
| **Gankyrin+CCL24** | 0.8145（0.6406-0.9138） | 0.8241（0.6432-0.9450） | 0.8697（0.7071-0.9623） | 0.7664（0.6833-0.8573） | 0.7544（0.6710-0.8487） | 0.7560（0.6737-0.8511） |
| **TNM stage+SSIGN** | 0.7825（0.6521-0.8929） | 0.7779（0.6461-0.8898） | 0.8086（0.6945-0.9226） | 0.7461（0.6454-0.8407） | 0.7210（0.6336-0.8313） | 0.7342（0.6431-0.8354） |
| **Gankyrin+CCL24+**  **TNM stage** | 0.8638（0.7377-0.9499） | 0.8752（0.7379-0.9621） | 0.9087（0.7859-0.9830） | 0.8361（0.7389-0.9102） | 0.8146（0.7109-0.8818） | 0.8219（0.7296-0.9034） |
| **Gankyrin+CCL24+**  **SSIGN** | 0.8610（0.7351-0.9465） | 0.8676（0.7267-0.9545） | 0.8909（0.7780-0.9898） | 0.8337（0.7356-0.9137） | 0.8115（0.7197-0.8914） | 0.8232（0.7282-0.9021） |
