# Supplementary material for: Blocking the autocrine regulatory loop of Gankyrin/STAT3/CCL24/CCR3 impairs the progression and pazopanib resistance of clear cell renal cell carcinoma
Source: Cell Death Dis. 2020 Feb 12;11(2):117. doi: 10.1038/s41419-020-2306-6 (PMC7015941; doi:10.1038/s41419-020-2306-6)
Supplement: Supplementary file 2 — Supplementary Figure Legend [file 41419_2020_2306_MOESM2_ESM.doc]

**Supplementary Figures and Figure Legends**

**Figure S1 Ectopic expression of gankyrin facilitates the proliferation, progression, pazopanib resistance, and tumorigenicity of ccRCC. a**, The expression of gankyrin in gankyrin overexpressed and control 786-O and 769-P cells was detected using western blot. **b**, The expression of gankyrin in IHC staining was evaluated by H-score, and the expression of Ki-67 in IHC staining was evaluated by the percentage of positive cells (P value: Wilcoxon test). All data represent the mean ±SD, **p* < 0.05, ***p* < 0.01 and ****p* < 0.001.

**Figure S2 Knockdown of gankyrin inhibits the proliferation, progression, pazopanib resistance, and tumorigenicity of ccRCC. a**, The expression of gankyrin in gankyrin knockdown and control 786-O and 769-P cells was detected using western blot. **b**, The expression of gankyrin in IHC staining was evaluated by H-score, and the expression of Ki-67 in IHC staining was evaluated by the percentage of positive cells (P value: Wilcoxon test). All data represent the mean ±SD, **p* < 0.05, ***p* < 0.01 and ****p* < 0.001.

**Figure S3 Gankyrin facilitates growth and progression of ccRCC cells through promoting the autocrine CCL24/CCR3. a-b,** Volcano plot of differential expressed genes from 786-O cells without or with gankyrin overexpressed, and gankyrin knockdown and control 786-O cells. **c,** The expression of CCL24 in CCL24 knockdown and control 786-O and 769-P cells was detected using western blot. **d,** CCK-8 assays were performed to determine the viability of 786-O or 769-P cells with or without gankyrin overexpression in the absence or presence of CCL24 knockdown or CCR3 knockdown at the indicated times. The data are presented as fold-changes relative to the control group. **e,** The percentage of apoptotic 786-O or 769-P cells with or without gankyrin overexpression in the absence or presence of CCL24 knockdown or CCR3 knockdown was analyzed by annexin V/PI double-staining and flow cytometry assays. **f-g,** Representative images and statistical analysis of the results from the invasion (f) and migration (g) assays of 786-O and 769-P cells with or without gankyrin overexpression in the absence or presence of CCL24 knockdown or CCR3 knockdown are presented (scale bar = 200 µm). **h,** Representative H&E and IHC staining for gankyrin and Ki-67 was performed in subcutaneous xenografts from the different groups (scale bar = 50 μm). The expression of gankyrin in IHC staining was evaluated by H-score, and the expression of Ki-67 in IHC staining was evaluated by the percentage of positive cells (P value: Wilcoxon test). **i,** STRING database (https://string-db.org/cgi/network.pl?taskId=he8ZsiRNWAQz) was used to predict the mostly credible functional partner of CCL24. **j,** Real-time PCR was performed to determine the expression of *CCR3* mRNA in 786-O or 769-P cells with or without gankyrin overexpression in the absence and presence of CCL24 knockdown. **k,** Western blot assays were used to detect the protein expression of p-Erk, Erk, p-Akt and Akt in 786-O or 769-P cells with or without gankyrin overexpression in the absence or presence of the CCL24 antibody (10 ng/ml) or SB328437 (10 ng/ml). **l,** The expression of CCR3 in CCR3 knockdown and control 786-O and 769-P cells was detected using western blot. All data represent the mean ±SD, **p* < 0.05, ***p* < 0.01 and ****p* < 0.001.

**Figure 4 CCL24 exerts a protumoral role in ccRCC, and high CCL24 expression in ccRCC patients predicts poor postoperative prognosis. a,** Kaplan-Meier analyses of the OS and PFS of ccRCC patients were performed with the combined cohort (n = 256) (p value: log-rank test). All the data are presented as the means ± SDs, **P* < 0.05, ***P* < 0.01 and ****P* < 0.001.

**Figure S5 Gankyrin/STAT3/CCL24/CCR3 forms a positive autocrine regulatory loop in ccRCC. a**, Western blot was used to detect the protein expression of STAT3 in 786-O or 769-P cells treated with STAT3 siRNAs (1^#^, 2^#^, and 3^#^). **b**, Real-time PCR was performed to determine the expression of *CCL24* mRNA in 786-O or 769-P cells with or without gankyrin knockdown in the absence and presence of STAT3 overexpression. c**,** The expression of STAT3 in STAT3 overexpressed and control 786-O and 769-P cells was detected using western blot. **d,** Real-time PCR was performed to determine the expression of *CCL24* mRNA in 786-O or 769-P cells with or without STAT3 overexpression. **e,** Real-time PCR was performed to determine the expression of *CCL24* mRNA in 786-O or 769-P cells with or without STAT3 knockdown. **f,** JASPAR software was used to predict putative STAT3-binding sites in the CCL24 promoter. **g**, Western blot assays were used to detect the protein expression of gankyrin, p-STAT3, and STAT3 in 769-P cells treated with human recombinant CCL24 protein (5 ng/ml) for 3 and 5 days in the absence or presence of SB328437 (10 ng/ml). **h**, Western blot assays were performed to detect the protein expression of gankyrin, p-STAT3, and STAT3 in 769-P cells treated with human recombinant CCL24 protein (3, 5 ng/ml) for 3 days in the absence or presence of SB328437 (10 ng/ml). **i**, Immunoprecipitation assays were employed to examine the binding of gankyrin to STAT3 in 769-P cells treated with human recombinant CCL24 protein (5 ng/ml) for 3 days in the absence or presence of SB328437 (10 ng/ml). All data represent the mean ±SD, **p* < 0.05, ***p* < 0.01 and ****p* < 0.001.

**Figure S6 Blocking the positive autocrine regulatory loop ameliorates pazopanib resistance and inhibits lung metastasis of ccRCC. a**, CCK8 assay of pazopanib-resistant 786-O (786-O-PR) and parental 786-O cells upon pazopanib treatment at indicated concentrations for 60 h. **b**, 786-O or 786-O-PR were respectively treated without or with pazopanib (5 μM) at different times and the viability of the cells was detected by CCK-8 assays. **c**, 786-O or 786-O-PR were respectively treated without or with pazopanib (5 μM) for 36 h and the resulting apoptosis was analyzed by annexin V/PI double-staining and flow cytometry assays. All data represent the mean ±SD, **p* < 0.05, ***p* < 0.01 and ****p* < 0.001.

**Figure S7 The combination of gankyrin, STAT3 or CCL24, and established indicators yields superior prognostic accuracy in predicting the prognosis of ccRCC patients. a,** Representative images of H&E staining and IHC staining for gankyrin and CCL24 in ccRCC tissues are presented (scale bar = 50 µm), and the results from the correlation analysis between gankyrin and CCL24 expression in the ccRCC samples are shown (n = 256, *r^2^* = 0.8392, *p <* 0.001). **b-c,** According to the H-scores for gankyrin and the percentage of positive cells for STAT3 in ccRCC specimens, the patients were divided into four groups. Kaplan-Meier analyses of the OS and PFS of ccRCC patients were performed with the validation (b, n = 128) and combined cohorts (c, n = 256) (p value: log-rank test). **d-e**, According to the H-scores for gankyrin and CCL24 in ccRCC specimens, the patients were divided into four groups. Kaplan-Meier analyses of the OS and PFS of ccRCC patients were performed with the validation (d, n = 128) and combined cohorts (e, n = 256) (p value: log-rank test). All the data are presented as the means ± SDs, **P* < 0.05, ***P* < 0.01 and ****P* < 0.001.
