# Supplementary figures and images for: Blocking the autocrine regulatory loop of Gankyrin/STAT3/CCL24/CCR3 impairs the progression and pazopanib resistance of clear cell renal cell carcinoma
Source: Cell Death Dis. 2020 Feb 12;11(2):117. doi: 10.1038/s41419-020-2306-6 (PMC7015941; doi:10.1038/s41419-020-2306-6)

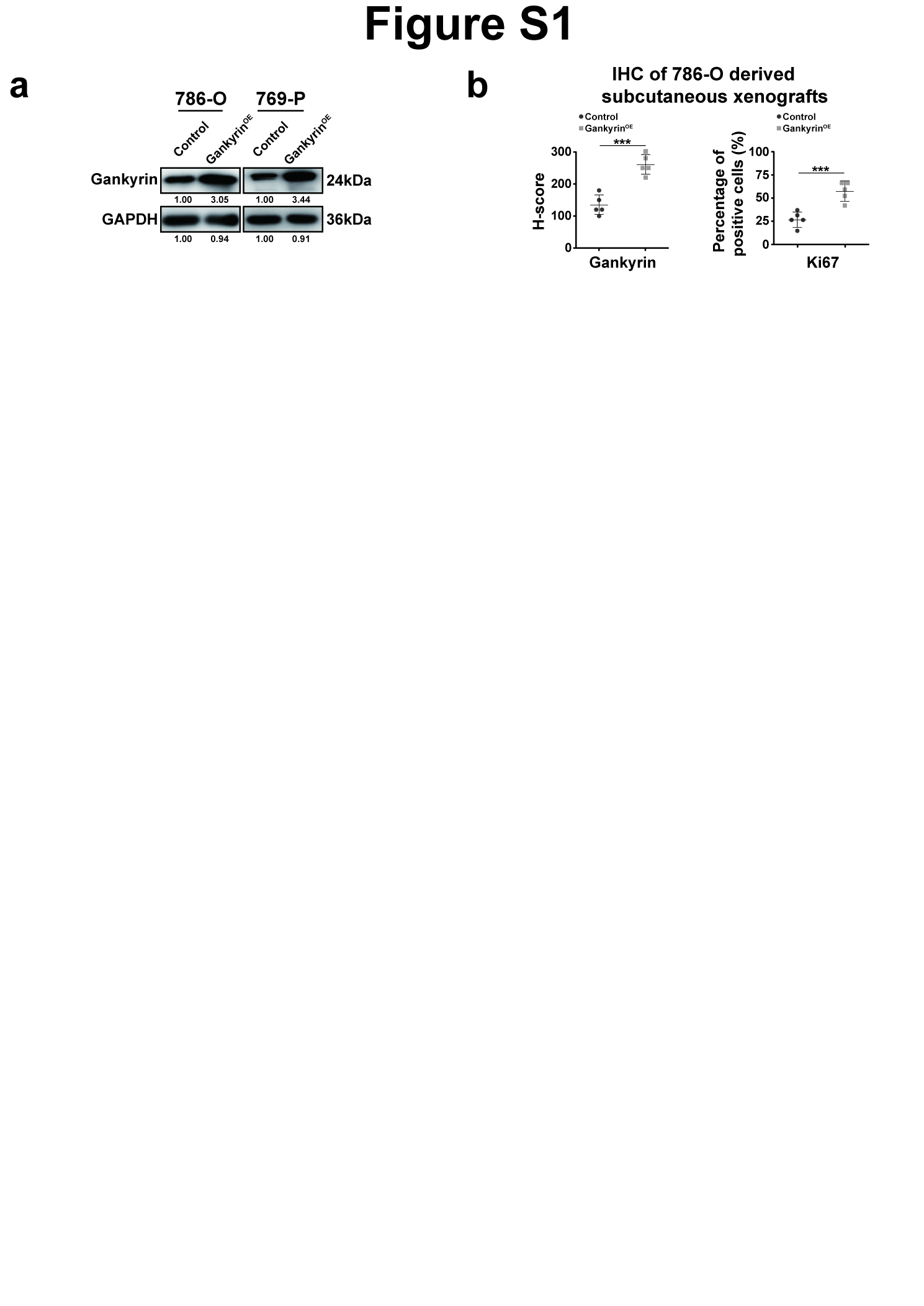

Supplement: Supplementary file 3 — Supplementary Figure S1 [file 41419_2020_2306_MOESM3_ESM.tif]

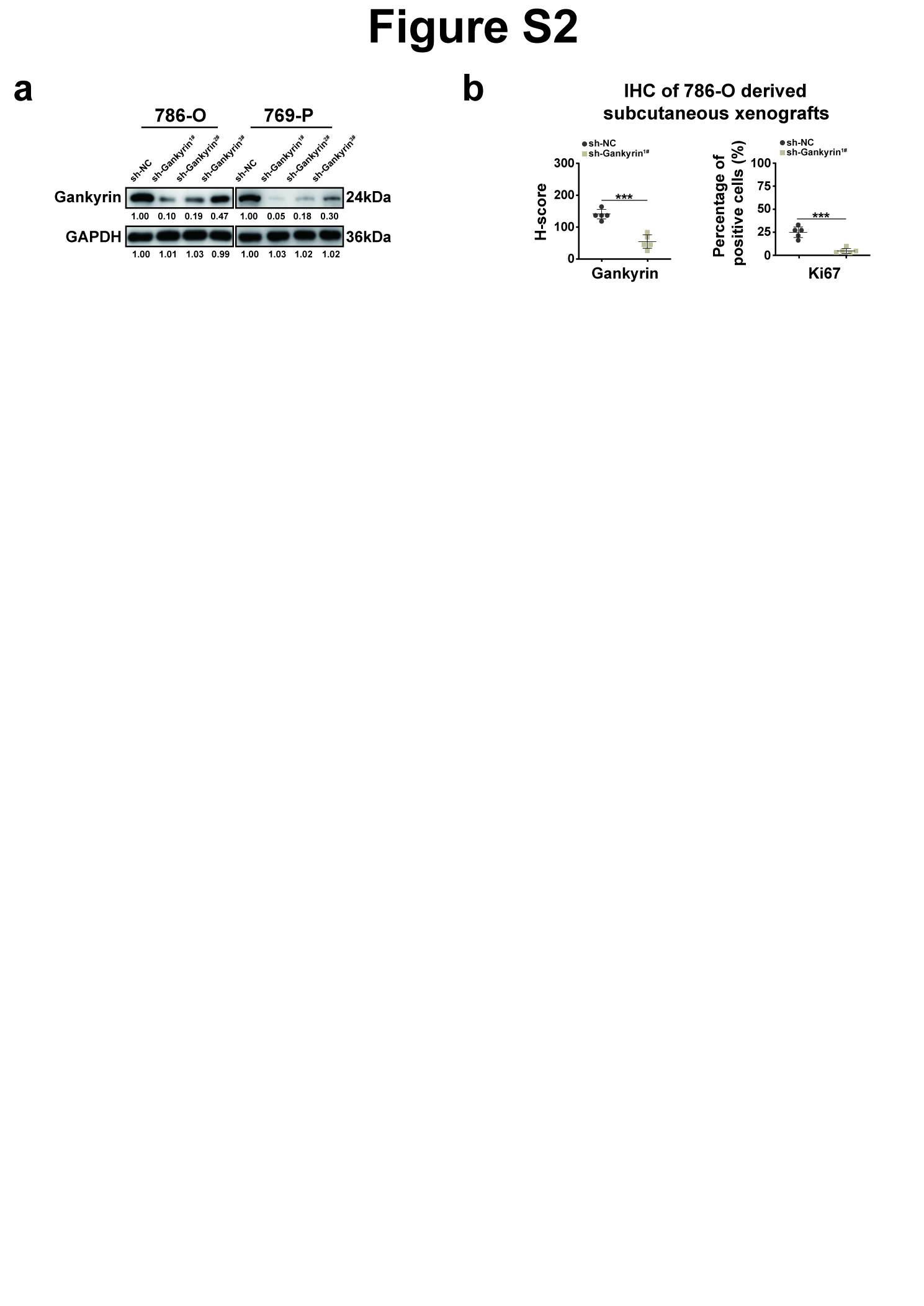

Supplement: Supplementary file 4 — Supplementary Figure S2 [file 41419_2020_2306_MOESM4_ESM.tif]

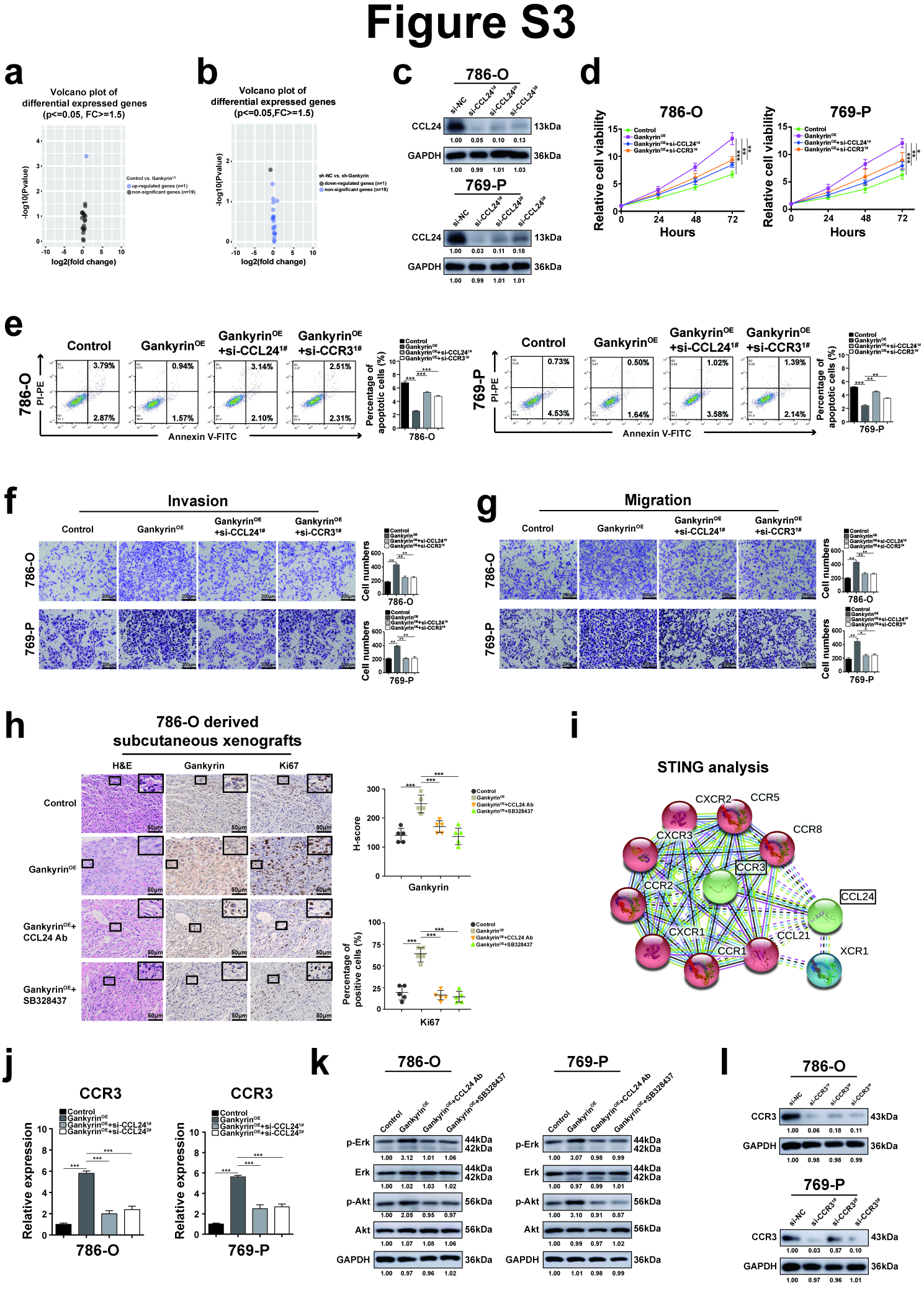

Supplement: Supplementary file 5 — Supplementary Figure S3 [file 41419_2020_2306_MOESM5_ESM.tif]

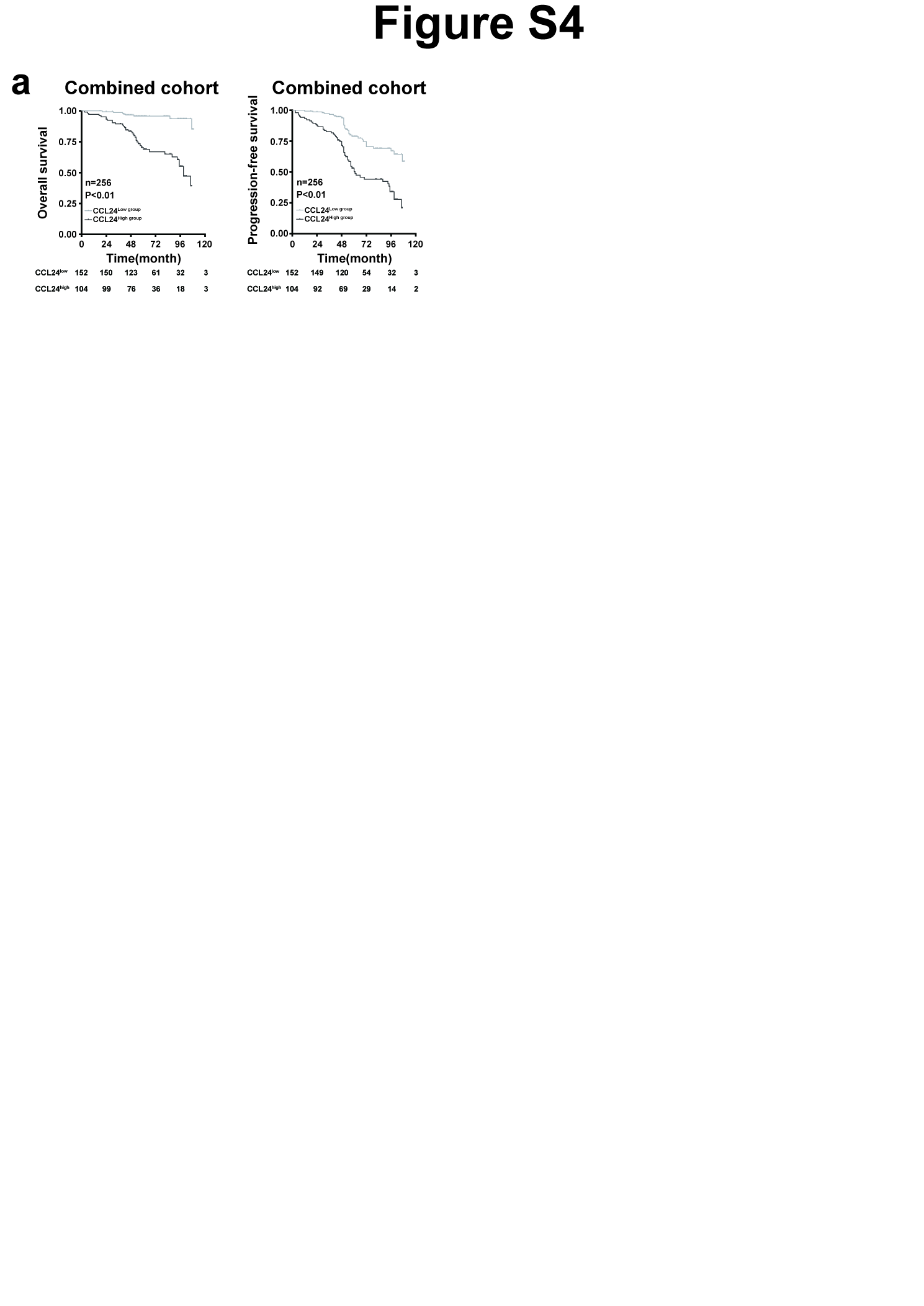

Supplement: Supplementary file 6 — Supplementary Figure S4 [file 41419_2020_2306_MOESM6_ESM.tif]

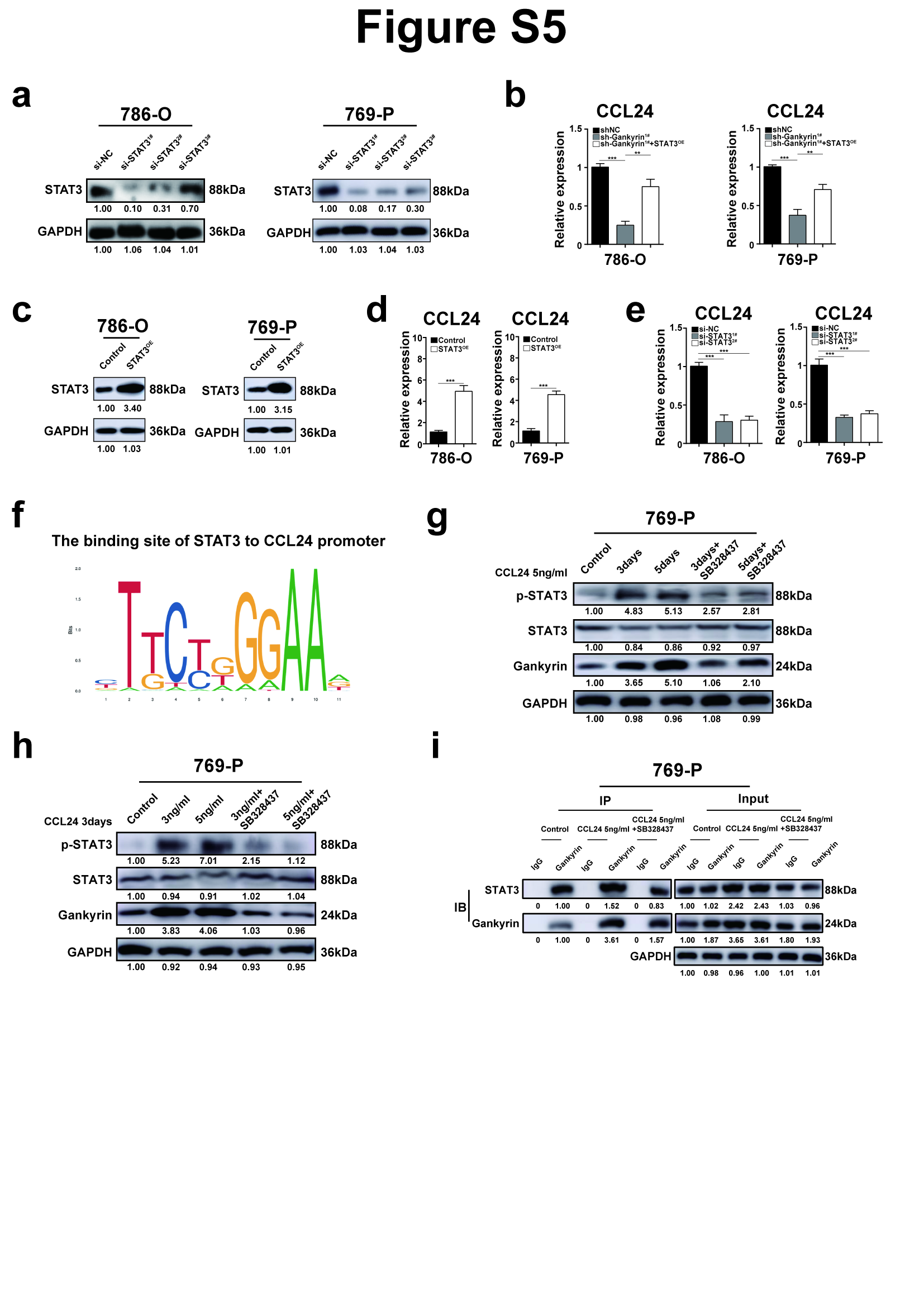

Supplement: Supplementary file 7 — Supplementary Figure S5 [file 41419_2020_2306_MOESM7_ESM.tif]

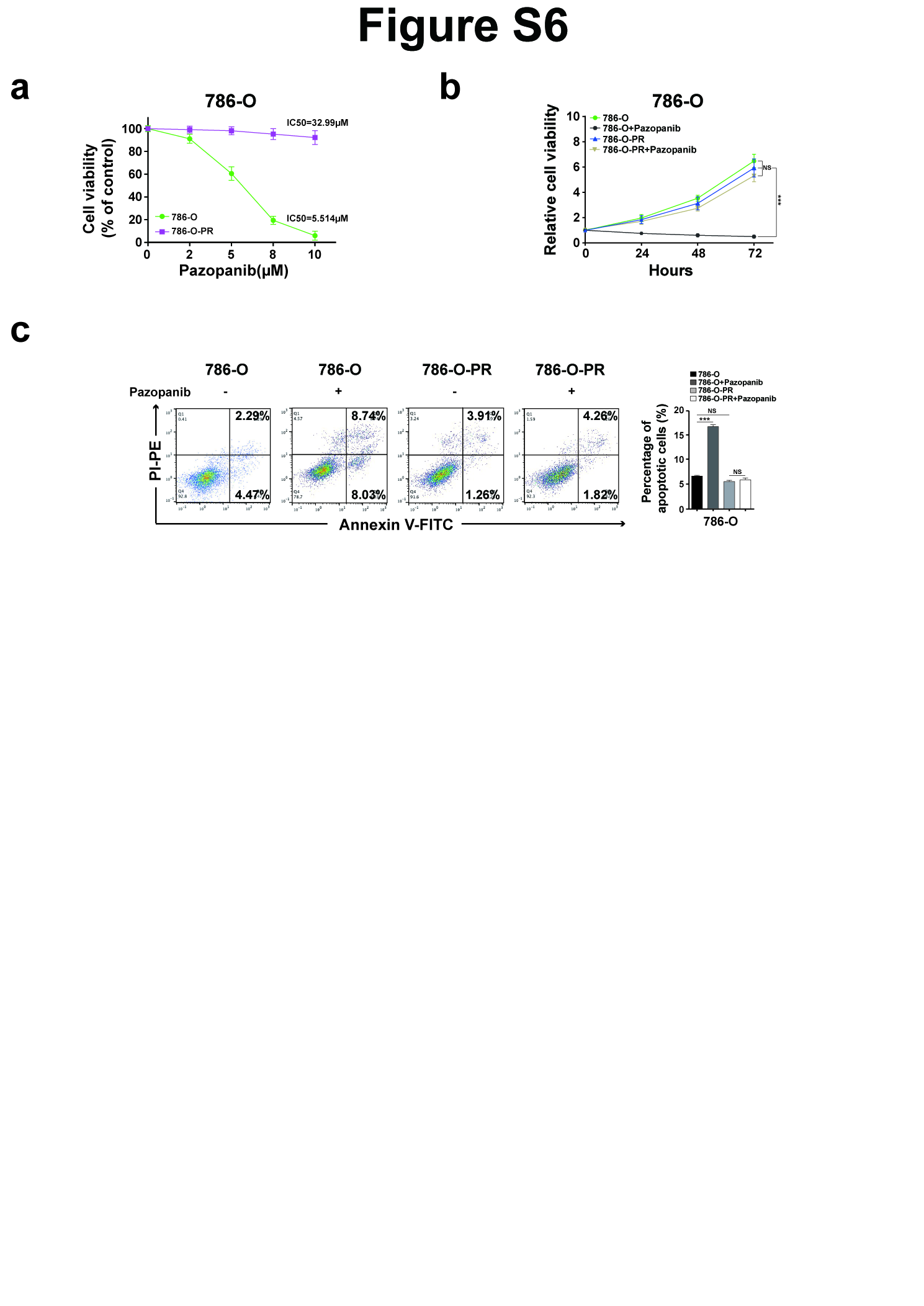

Supplement: Supplementary file 8 — Supplementary Figure S6 [file 41419_2020_2306_MOESM8_ESM.tif]

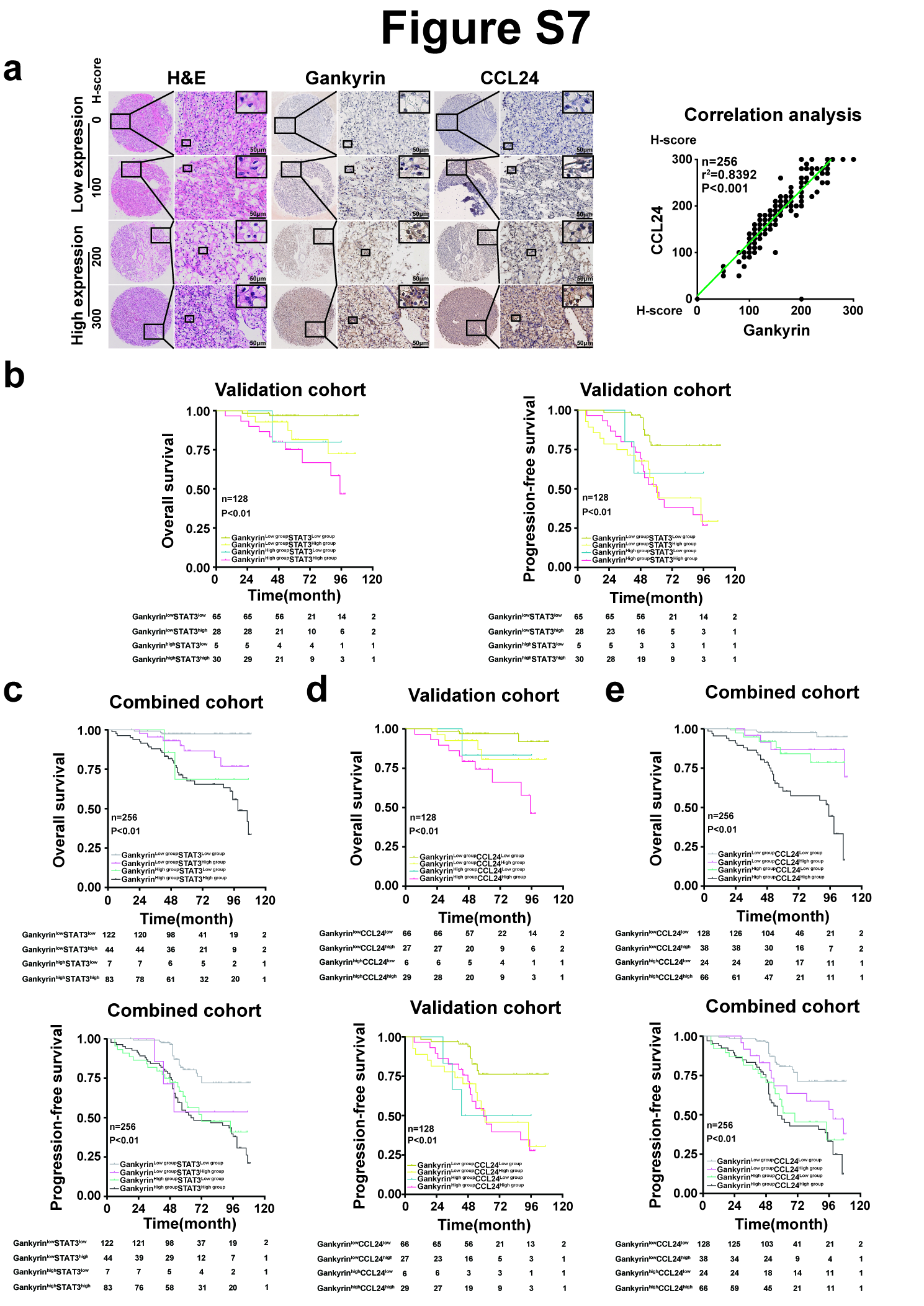

Supplement: Supplementary file 9 — Supplementary Figure S7 [file 41419_2020_2306_MOESM9_ESM.tif]
